# A novel therapeutic antibody screening method using bacterial high-content imaging reveals functional antibody binding phenotypes of *Escherichia coli* ST131

**DOI:** 10.1038/s41598-020-69300-8

**Published:** 2020-07-24

**Authors:** Mailis Maes, Zoe A. Dyson, Sarah E. Smith, David A. Goulding, Catherine Ludden, Stephen Baker, Paul Kellam, Stephen T. Reece, Gordon Dougan, Josefin Bartholdson Scott

**Affiliations:** 1 Department of Medicine, Cambridge Institute for Therapeutic Immunology & Infectious Disease, University of Cambridge, Cambridge, UK; 2 Department of Infectious Diseases, Central Clinical School, Monash University, Melbourne, VIC 3004 Australia; 3 London School of Hygiene and Tropical Medicine, London, UK; 4 Kymab Ltd, Babraham Research Campus, Cambridge, UK; 5 Wellcome Sanger Institute, Hinxton, UK; 6 Department of Infectious Disease, Imperial College London, London, UK

**Keywords:** Bacteria, Pathogens, Antibody therapy, High-throughput screening, Phenotypic screening, Microbiology, Antimicrobial resistance

## Abstract

The increase of antimicrobial resistance (AMR), and lack of new classes of licensed antimicrobials, have made alternative treatment options for AMR pathogens increasingly attractive. Recent studies have demonstrated anti-bacterial efficacy of a humanised monoclonal antibody (mAb) targeting the O25b O-antigen of *Escherichia coli* ST131. To evaluate the phenotypic effects of antibody binding to diverse clinical *E. coli* ST131 O25b bacterial isolates in high-throughput, we designed a novel mAb screening method using high-content imaging (HCI) and image-based morphological profiling to screen a mAb targeting the O25b O-antigen. Screening the antibody against a panel of 86 clinical *E. coli* ST131 O25:H4 isolates revealed 4 binding phenotypes: no binding (18.60%), weak binding (4.65%), strong binding (69.77%) and strong agglutinating binding (6.98%). Impaired antibody binding could be explained by the presence of insertion sequences or mutations in O-antigen or lipopolysaccharide core biosynthesis genes, affecting the amount, structure or chain length of the O-antigen. The agglutinating binding phenotype was linked with lower O-antigen density, enhanced antibody-mediated phagocytosis and increased serum susceptibly. This study highlights the need to screen candidate mAbs against large panels of clinically relevant isolates, and that HCI can be used to evaluate mAb binding affinity and potential functional efficacy against AMR bacteria.

## Introduction

Antimicrobial resistance (AMR) is one of the greatest current challenges in global health^1^. Increasing AMR and a lack of antimicrobials in the pharmaceutical pipeline makes alternative therapeutic approaches increasingly important. Passive antibody transfer has historically been used to treat bacterial infections, such as diphtheria, tetanus, and pneumococcal pneumonia^2,3^, making antibodies a potential therapeutic approach. Many biologics, including monoclonal antibodies (mAbs) are being used increasingly in oncology, autoimmune diseases, and the prevention of some viral infections^4,5^. However, mAbs have been found to have limited utility against bacteria^6^. This lack of effectiveness is, in part, because bacterial species are generally antigenically diverse and conserved immunogenic surface components can be masked by large structures such as capsules. Identifying tractable therapeutic antibody targets that are generic or specific for particular pathogenic or AMR bacteria would be a valuable addition to our current arsenal of therapeutic options.

Numerous globally dispersed clades of pathogenic bacteria associated with broad AMR phenotypes have emerged in recent decades^7–9^. One example is the ST131 O25b:H4 clonal group of *Escherichia coli* that is characterised by the acquisition of extended-spectrum beta-lactamases (ESBLs) and fluoroquinolone resistance^10^. Notably, *E. coli* ST131 O25b:H4 have a specific O-antigen, which is potentially an attractive target for mAbs. The humanised monoclonal antibody, 3E9-11, specifically targeting this O25b O-antigen has recently demonstrated promising efficacy^11^. This antibody, which is in preclinical development, exhibits multiple modes of antibacterial activity and exhibited protection in mice^11,12^. Mutations in the O-antigen region have previously been shown to affect serum resistance and thus clinical outcome^13–16^. Therefore for an O-antigen antibody to be of clinical utility it is important to demonstrate that these anti-bacterial activities function against a diverse collection of *E. coli* ST131 O25b associated with disease in healthcare settings.

High-content imaging (HCI) is a powerful phenotypic screening approach that combines high-throughput automated microscopy with comprehensive image analysis to quantify multiple morphological and functional cellular features. This type of image-based morphological profiling can be used for high-throughput screening of drugs, simultaneously evaluating potency as well as mode-of-action^17,18^. HCI has been predominantly applied to mammalian cells and tissue where the examined variables include cell and organelle shape, signal transduction, gene expression and metabolism. In addition to studying mammalian cells, HCI has also been used to study intracellular pathogens such as *Mycobacterium tuberculosis*^19,20^, and more recently individual bacteria under drug exposure^21^. HCI has now reached a point where populations of bacteria can be phenotyped at single cell resolution in high-throughput to enable the simultaneous screening of multiple isolates of diverse bacterial clades.

Here we designed a novel mAb screening method using HCI and image-based morphological profiling to measure the antimicrobial potential of a variant of 3E9-11, which targets the O-antigen of *E. coli* ST131 O25b:H4. We profiled 86 *E. coli* ST131 O25b:H4 clinical isolates in imaging assays at a level of resolution that identified individual bacteria in 96 well plates. Our analysis revealed distinct mAb binding phenotypes within the *E. coli* ST131 O25b:H4 population that were directly associated with mAb function.

## Results

### Screening antibodies against bacteria using high-content imaging

**Figure 1:**
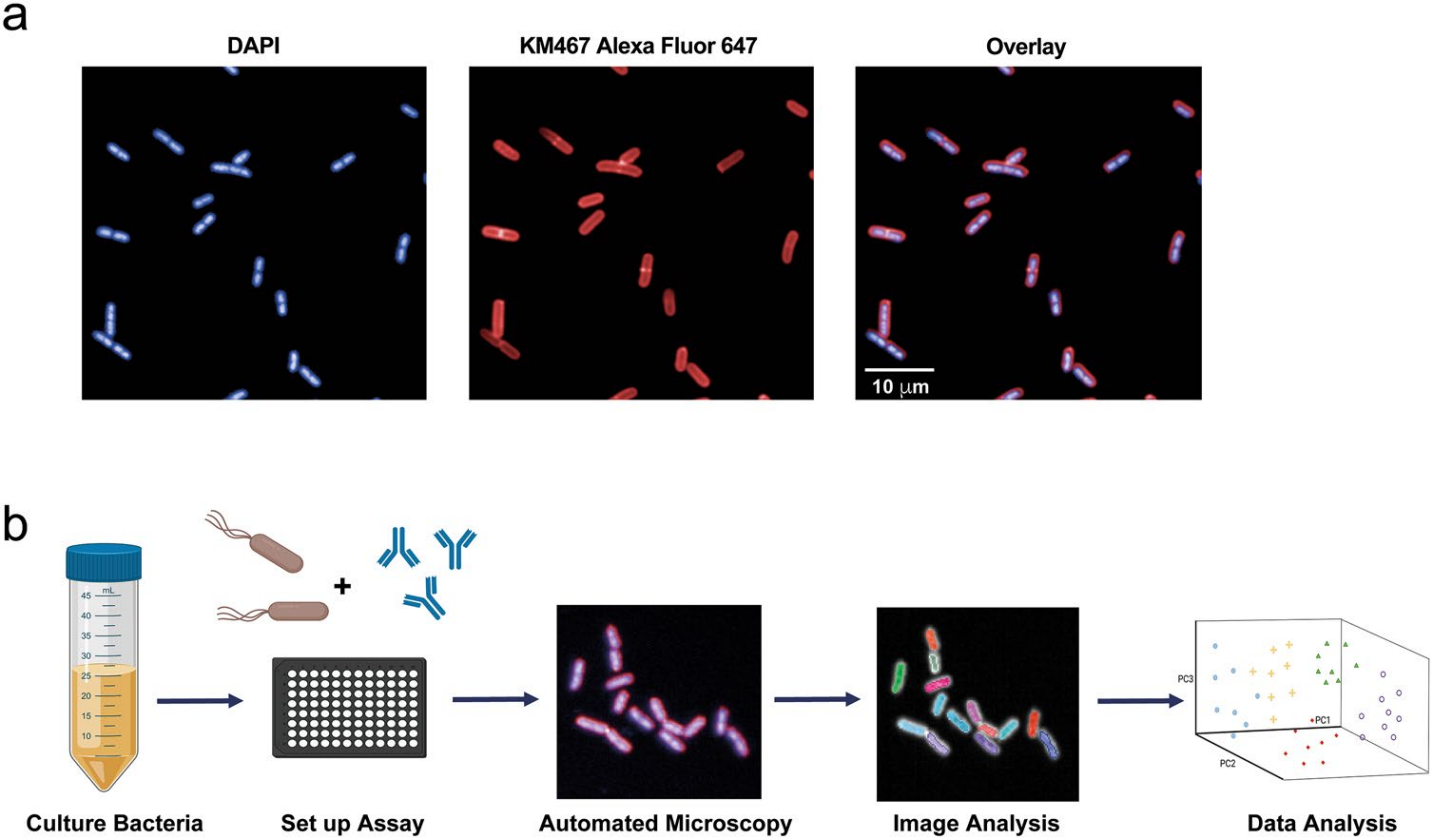
High-content imaging to screen mAbs against bacteria*. E. coli* ST131 NCTC13441 were stained with DAPI (nucleic acid stain) and KM467 followed by an Alexa Fluor 647-conjugated secondary antibody (**a**). Bacterial high-content imaging workflow (**b**): bacterial overnight cultures were diluted and added to microtiter plates and left to adhere for 2 h at 37 °C. Plates were fixed, and incubated with KM467 for 1 h, followed by an Alexa Fluor 647-conjugated secondary antibody and DAPI for 30 min. The plates were imaged on the Opera Phenix using a 63 × water immersion objective, and the images were analysed using the Harmony software. Data was exported into R for further analysis. The schematic was created using icons from BioRender.com.

To evaluate HCI as a method for screening candidate mAbs against large panels of clinical isolates, whilst simultaneously determining the diagnostic and functional potentials of the antibody, we synthesised KM467, an IgG1 antibody based on the VH and VL sequences of 3E9-11, which specifically targets the O25b O-antigen of *E. coli* ST131. KM467 was tested for the ability to bind lipopolysaccharide (LPS) isolated from the *E. coli* ST131 O25b reference strain NCTC13441 using ELISA (Supplementary Fig. S1), and direct binding to whole bacteria was tested using the Perkin Elmer Opera Phenix high-content confocal microscope (Fig. 1a). KM467 recognized the target in both assays: exhibiting a clear titration curve in the ELISA and a strong staining pattern of the bacterial surface by confocal imaging.

**Figure 2:**
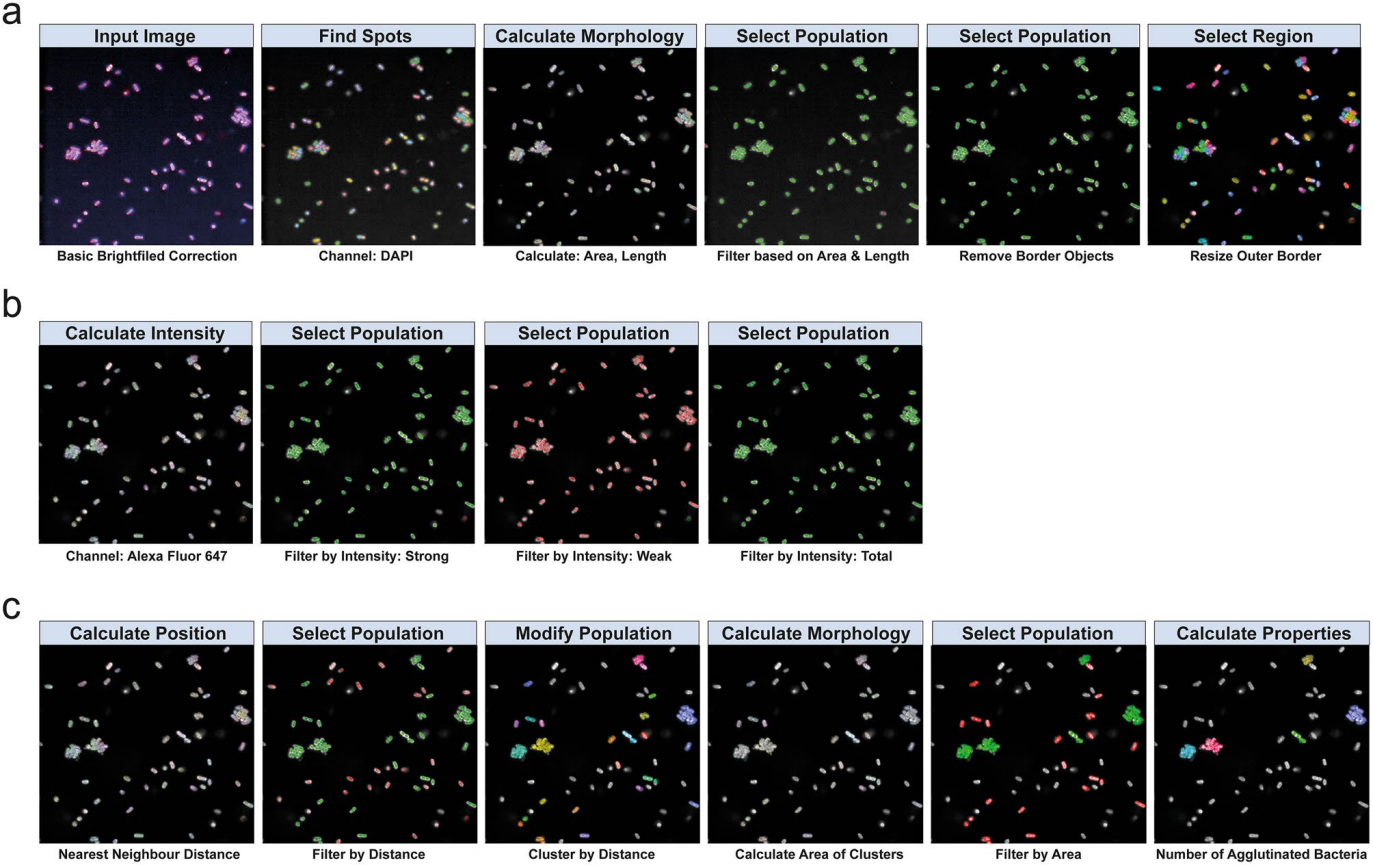
Antibody binding image analysis. Images were segmented in Harmony using the Find Spots building block and size filters were applied to distinguish individual bacteria and the outer border of each bacteria was resized to maximize antibody stain coverage (**a**). Intensity properties were calculated based on Alexa Fluor 647 intensity (antibody binding) and three sub-populations were defined based on strong, weak and total Alexa Fluor 647 intensity (**b**). Agglutination was defined based on bacterial position properties: nearest neighbour distance was used to filter and define bacterial clusters (**c**). Clusters were further filtered based on area to define true agglutination, finally, the number of agglutinating bacteria was calculated.

A bacterial antibody high-content screening workflow was developed for higher throughput screening as outlined in Fig. 1b. For bacterial imaging, overnight liquid cultures of *E. coli* NCTC13441 were diluted and added to ultra-thin, flat-bottom 96 well plates and left to adhere for 2 h at 37 °C. Bacteria were fixed with paraformaldehyde and incubated with KM467. Finally, bacteria were stained with DAPI and an Alexa Fluor 647-conjugated anti-human IgG secondary antibody in situ (Fig. 1a). The plates were imaged on an Opera Phenix using a 63 × water immersion objective and the images were analysed using the Harmony software. A full 96 well plate with 16 fields and 3 Z-stacks per well took 1 h to image, and the images displayed clearly defined individual bacteria (Fig. 1a). Images were segmented using the DAPI channel and the Harmony software ‘Find Spots’ building block, then size filters were applied to distinguish individual bacteria. A small outer border around each bacterium was included to identify Alexa Fluor 647 bound around the bacterial cell surface (Fig. 2a), and the Alexa Fluor 647 intensity was calculated for each segmented bacterium (Fig. 2b). An average of 2 × 10^3^ bacteria per well were analysed using this approach.

A KM467 titration curve was performed using the NCTC13441 O25b reference strain, as well as an *E. coli* ST131 isolate with O16 O-antigen (VRES0673) (Supplementary Fig. S2). No KM467 binding was observed to the O16 isolate, confirming specificity to the O25b O-antigen. The titration curve generated using image analysis (Supplementary Fig. S2b) correlated with the standard curve from the ELISA (Supplementary Fig. S1), and demonstrated that the optimal concentration of KM467 was 1 µg/ml. Bacterial imaging and antibody binding were found to be reproducible with no significant background from the secondary antibody in the negative control or when using the *E. coli* O16 isolate (Supplementary Fig. S2).

### Screening KM467 against clinical *E. coli* ST131 isolates revealed four distinct binding phenotypes

**Figure 3:**
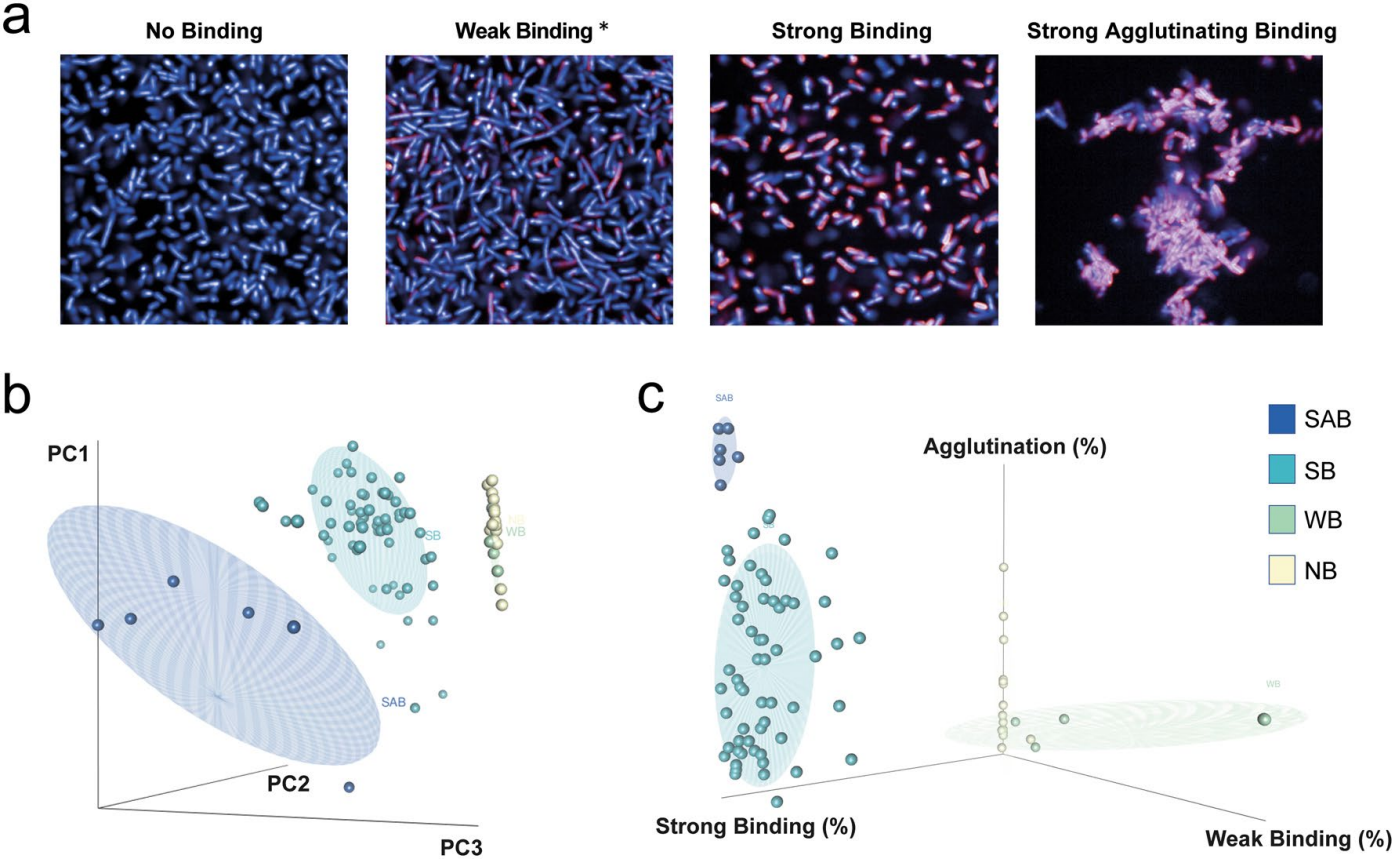
Screening KM467 against clinical *E. coli* ST131 isolates revealed four distinct binding phenotypes. Four antibody binding phenotypes were observed (**a**): no binding, weak binding, strong binding and strong agglutinating binding. *Over-exposed compared to other images. Principal Component Analysis distinguishing the different binding phenotypes (**b**) and a graph separating the phenotypes based on percentage weak binding, strong binding and agglutination (**c**) were generated in R.

KM467 was screened against a panel of 86 *E. coli* ST131 O25:H4 clinical isolates (Supplementary Table S1) in 96 well format and images were captured on the Opera Phenix and segmented using the Perkin Elmer Harmony software as described above (Fig. 2a–c, ãmentary Table S2). This screen revealed four distinct binding phenotypes (Fig. 3a) that were classified as: no binding (NB), weak binding (WB), strong binding (SB) and strong agglutinating binding (SAB). To confirm that the weak binding was not an artefact, titrated antibody was tested against representative isolates for each of the phenotypic classes (Supplementary Fig. S3). Although the percentage binding observed for the weak binders was much lower than that observed for the SB and SAB isolates, weak binding was genuine, as evidenced by increased binding observed with higher mAb concentrations and no binding observed with the secondary antibody only. The NB isolates displayed no observable KM467 binding at any concentration, even at high exposure (Supplementary Fig. S3). Based on the Alexa Fluor 647 intensities observed for the different phenotypes, intensity thresholds could be determined to differentiate between weak and strong antibody binding (Fig. 2b).

For efficient image segmentation, the bacterial density in the well needed to be optimal so that bacteria were sufficiently separated. This was particularly challenging for the agglutinating bacteria that were tightly clustered together. Conversely, this challenge formed the basis for measuring agglutination, allowing for automated identification of the SAB phenotype. To perform this analysis, the segmented bacterial positional properties were calculated: i.e. measuring distance to the “nearest neighbour” and by defining agglutination by cluster area and the quantification of bacteria within clusters (Fig. 2c, Supplementary Table S2). This approach, in combination with intensity measurements, convincingly separated the four phenotypes by principal component analysis (PCA) (Fig. 3b). The WB isolates clustered closely with the NB, but the SB isolates separated markedly from the WB and NB, and SAB isolates could be clearly distinguished from the non-agglutinating phenotypes. In addition, the four phenotypes could be effectively separated based on the proportions showing weak binding, strong binding and agglutination (Fig. 3c). Out of the 86 isolates 16 exhibited no KM467 binding (18.60%), 4 WB (4.65%), 60 SB (69.77%) and 6 SAB (6.98%) (Supplementary Table S1, Supplementary Table S3). One isolate (ECO0237) agglutinated both in the presence and absence of antibody but grouped with the SB isolates in all analyses, therefore this isolate was classified as SB since the antibody did not induce the agglutination. These results demonstrate that HCI can be used for high-throughput antibody screening and found that a single antibody can induce distinct *E. coli* ST131 isolate-specific phenotypic effects due to binding.

### Differences in O-antigen correlate with binding phenotypes

**Figure 4:**
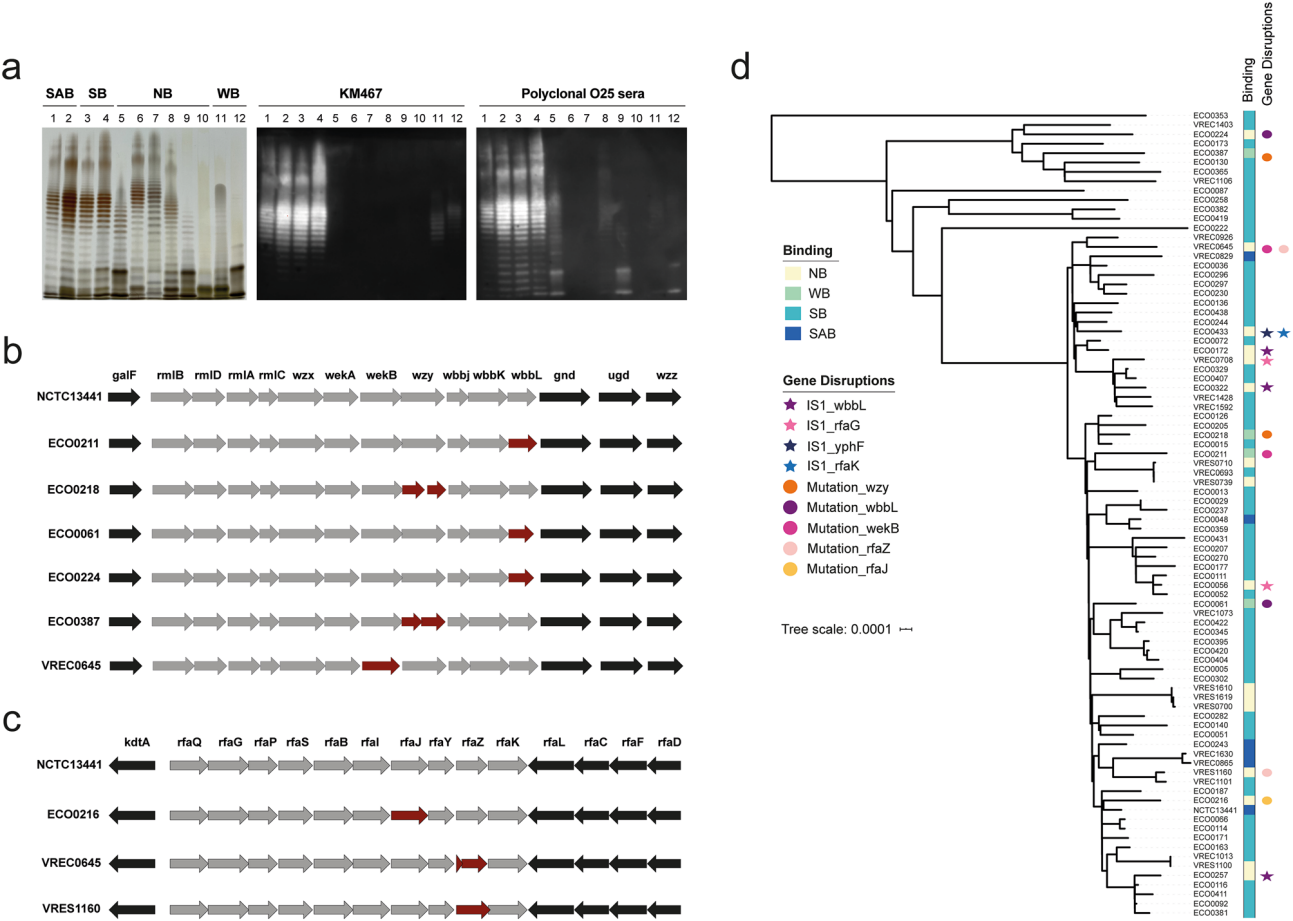
O-antigen integrity influences binding phenotypes. Isolated LPS was analysed by gel electrophoresis followed by silver staining (left) and immunoblotting using KM467 (middle) or O25 polyclonal antisera (right) (**a**). Lanes 1–12: NCTC13441, VREC0829, VREC1073, VREC1403, VREC0708, VRES1100, VRES1160, VRES1610, ECO0056, ECO0172, ECO0061, ECO0218. Binding phenotypes are indicated above the gel (lane 1–2 are SAB, lane 3–4 are SB, lane 5–10 are NB and lane 11–12 are WB). The O25b O-antigen genes (**b**) and LPS core genes (**c**) of NCTC13441 and a selection of isolates that did not bind KM467 were aligned using Prokka annotated fasta files and plotted using EasyFig2.2.2. Genes affected by mutations are highlighted in red. A core genome maximum likelihood phylogenetic tree of the *E. coli* ST131 O25b isolates, created using RAxML v8.2.9 and visualised using iTOL version 5.5.1 (https://itol.embl.de), was aligned with the antibody binding phenotypes and presence of mutations (circles) or IS*1* (stars) (**d**).

To investigate the different binding phenotypes, LPS was extracted from 35 representative isolates including the NCTC13441 reference. Silver staining revealed that a lack of binding or WB appeared to be associated with a lack of O-antigen, reduced O-antigen production or differing O-antigen lengths compared to the conserved O-antigen of the binders (Fig. 4a, and Supplementary Fig. S4). We could observe no obvious differences between the SB and the SAB organisms.

A selection of LPS representing each binding phenotype was further analysed by immunoblotting using KM467 and polyclonal anti-O25 typing sera (Fig. 4a). Immunoblotting using KM467 correlated with the HCI data, with strong bands only observed for those isolates with SB or SAB binding phenotypes. Isolates classified as WB by HCI also had weak staining by immunoblotting with KM467; however, their banding pattern was distinct from that of the SB and SAB isolates. The polyclonal anti-O25 sera proficiently stained the O-antigen of the SB and SAB isolates, but also showed weaker binding to some of the isolates that showed weak or no KM467 binding. Notably, the NB isolates with higher molecular weight O-antigen exhibited no binding even in the presence of the polyclonal sera. These results show that the inability of KM467 to bind some isolates is associated with differences in or lack of O-antigen.

### Correlating genome content with phenotype

To address the observed differences in O-antigen in terms of genetics, we obtained whole genome sequence data for each isolate in our study and analysed the read data using SRST2^22^ in combination with an *E. coli *in silico serotyping scheme^23^. This analysis confirmed that all 86 isolates were genotypically serotype O25b (Supplementary Table S1). Raw read data were assembled with Unicycler^24^ and alignments of the O-antigen gene cluster and flanking genes (*galF, rmlB, rmlD, rmlA, rmlC, wzx, wekA, wekB, wzy, wbbj, wbbK, wbbL, gnd, ugd, wzz*) from each isolate were extracted manually to identify any genetic variation in this region (Fig. 4b, Table 1, Supplementary Fig. S5).

**Table 1:** O-antigen and LPS core genes disrupted by mutations or *IS*1.

Isolate	Binding phenotype	Disrupted gene	Gene function	Mutation	Insertion
ECO0056	NB	rfaG	Glucosyltransferase (LPS core biosynthesis)	–	IS1
ECO0172	NB	wbbL	Rhamnosyltransferase (O-antigen biosynthesis)	–	IS1
ECO0216	NB	rfaJ	1,2-glucosyltransferase (LPS core biosynthesis)	Missense	–
ECO0224	NB	wbbL	Rhamnosyltransferase (O-antigen biosynthesis)	Nonsense	–
ECO0257	NB	wbbL	Rhamnosyltransferase (O-antigen biosynthesis)	–	IS1
ECO0322	NB	wbbL	Rhamnosyltransferase (O-antigen biosynthesis)	–	IS1
EOO0433	NB	yphF	Rhamnose ABC transporter	–	IS1
EOO0433	NB	rfaK	Heptosyltransferase (LPS core biosynthesis)	–	IS1
VREC0645	NB	wekB	Putative glycosyltransferase	Nonsense	–
VREC0645	NB	rfaZ	Lipopolysaccharide core biosynthesis protein	Frameshift	–
VREC0708	NB	rfaG	Glucosyltransferase (LPS core biosynthesis)	–	IS1
VRES1160	NB	rfaZ	Lipopolysaccharide core biosynthesis protein	Frameshift	–
ECO0061	WB	wbbL	Rhamnosyltransferase (O-antigen biosynthesis)	Nonsense	–
ECO0211	WB	wbbL	Rhamnosyltransferase (O-antigen biosynthesis)	Missense	–
ECO0218	WB	wzy	O-antigen polymerase	Frameshift	–
ECO0387	WB	wzy	O-antigen polymerase	Frameshift	–

Two of the WB isolates, ECO0218 and ECO0387, harboured frameshift mutations resulting in a split of *wzy* into two open reading frames. The *wzy* gene encodes an O-antigen polymerase that regulates O-antigen chain length , and disruption of this gene may explain reduced KM467 binding. This observation is consistent with the lack of O-antigen observed by silver staining (Fig. 4a lane 12, Supplementary Fig. S4 lanes 27 and 30). A third WB isolate (ECO0061) and one NB isolates (ECO0224) had a truncated *wbbL* gene which encodes a rhamnosyltransferase. In addition, the fourth WB isolate (ECO0211) had a missense mutation in the same gene resulting in a change from glycine to glutamic acid at codon 60 (G60E). These mutations likely impact both O-antigen integrity and the antibody binding affinity as each O25b O-antigen repeat contains both rhamnose and *O*-acetyl-rhamnose^25^. A second NB isolate (VREC0645) had a premature stop codon in the putative glycosyltransferase *wekB*. The remaining 10 isolates that displayed no binding to the antibody appeared to have intact O-antigen operons, despite having atypical O-antigen repeat patterns.

To further investigate the lack of KM467 binding to these NB isolates, hybrid short and long-read assemblies from one SAB, one SB and three NB isolates were aligned using Mauve^26^, and inspected manually to identify potential inversions and indels in the O-antigen, LPS core and capsule biosynthesis regions. Insertion sequence IS*Ec10* was detected upstream of *kpsF* and IS*1* downstream of *kpsT* in the capsule biosynthesis regions of the NCTC13441 reference isolate, and subsequently we screened for the presence of IS*Ec10* and IS*1* using ISmapper^27^ in all genomes. The presence of IS*Ec10* upstream of *kpsF* did not appear to correlate with binding phenotype, nor was this type of IS found in any other relevant genomic regions (Supplementary Table S4). In addition, the presence of IS*1* downstream of *kpsT* was only observed in the NCTC13441 reference isolate that had an SAB antibody binding phenotype. However, IS*1* was present in three NB isolates in *wbbL* (ECO0172, ECO0257 and ECO0322), and in two NB isolates (ECO0056 and VREC0708) in *rfaG* encoding a glucosyltransferase involved in LPS core biosynthesis (Table 1). Mutations in *rfaG* have previously been associated with a rough LPS phenotype^28^. However, both these isolates displayed some shorter O-antigen repeating units, indicating that RfaG was not completely inactive (Fig. 4a lanes 5 and 9, Supplementary Fig. S4 lanes 11 and 16). ECO0433 had an IS*1* in rhamnose ABC transporter gene *yphF,* which could potentially affect the rhamnose composition of the O-antigen, and also an IS*1* in *rfaK,* which encodes a heptosyltransferase involved in core biosynthesis, which may explain the lack of O-antigen observed by LPS gel electrophoresis (Supplementary Fig. S4, lane 35).

Since IS*1* was found in genes affecting the LPS core, the core biosynthesis gene cluster (*kdtA, rfaQ, rfaG, rfaP, rfaS, rfaB, rfaI, rfaJ, rfaY, rfaZ, rfaK, rfaL, rfaC, rfaF, rfaD*) was extracted manually from assemblies and analysed by multiple sequence alignment (Fig. 4c, Table 1, Supplementary Fig. S5), and mutations were identified in two additional genes. NB isolate ECO0216 had a mutation resulting in a premature stop codon in *rfaJ* which encodes a 1,2-glucosyltransferase, and VREC0645 and VRES1160 (both NB) had frameshift mutations in *rfsZ* which is involved in 3-deoxy-D-manno-oct-2-ulosonic acid (Kdo) III attachment in the inner LPS core^29^. Although the core bands appear to be affected in both isolates, interestingly, VRES1160 displayed longer O-antigen repeats that were not detected by the polyclonal anti-O25 sera (Fig. 4a lane 7). In total, the identified mutations and the presence of IS*1* accounted for 10 of 16 NB and all four of the WB isolates.

A maximum-likelihood phylogenetic tree was inferred from core genome SNP alignments of the study isolates. Phenotypic binding data and the presence of mutations in the O-antigen or core biosynthesis region and presence of IS*1* within these was overlaid as metadata to observe any potential relationship between phenotype and genotype (Fig. 4d). There was no relationship between phylogenetic cluster and KM467 binding phenotype in this data set, and gene disruptions were associated with mutation and recombination events in individual isolates.

### Antibody-induced phagocytosis is more efficient in agglutinating isolates

**Figure 5:**
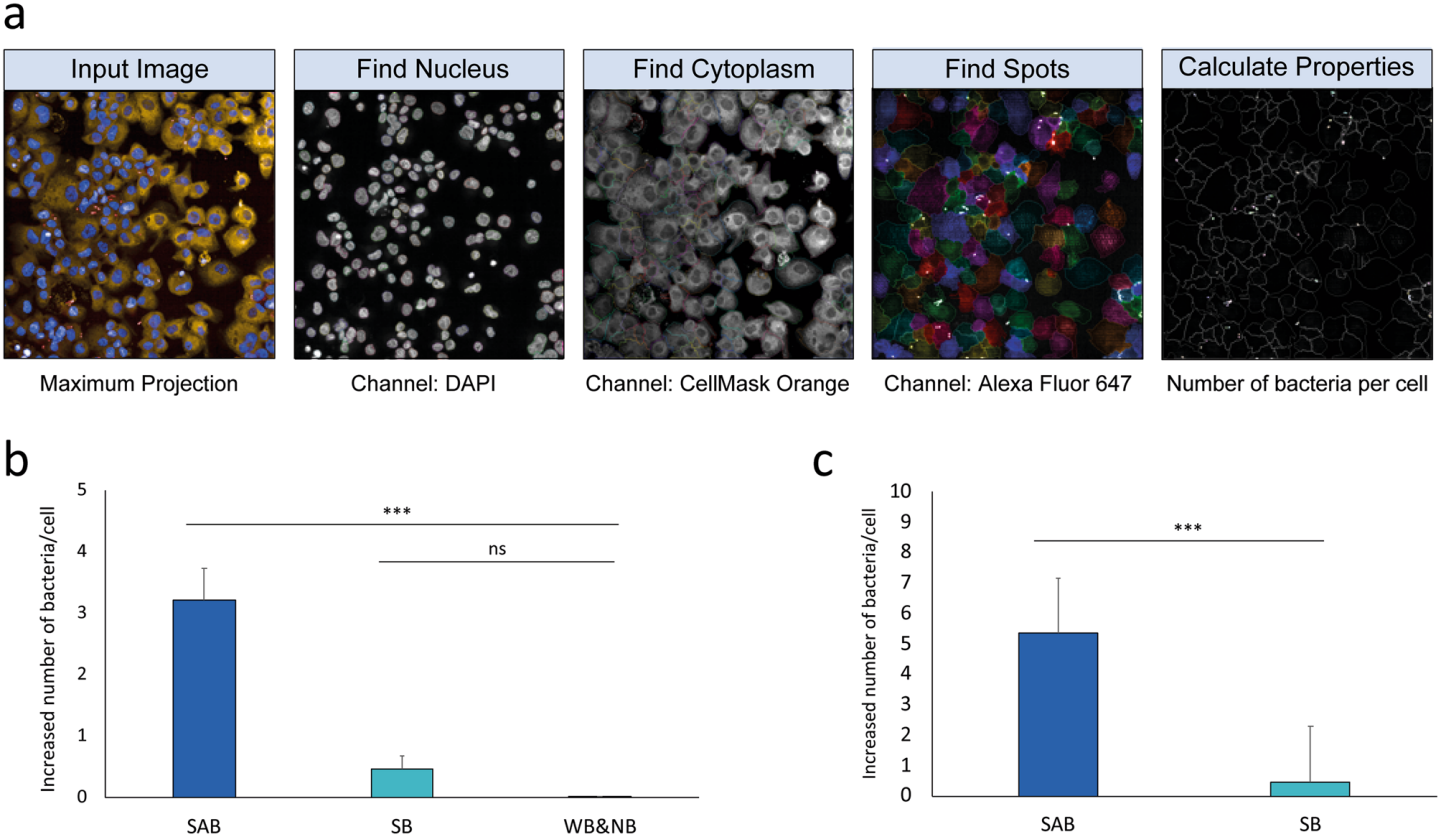
Clearance by phagocytosis and opsonophagocytosis is directly linked to binding phenotype. Macrophage phagocytosis and opsonophagocytosis imaging and analysis (**a**): cells were stained with CellMask Orange (cytoplasm) and DAPI (nucleic acid) and bacteria were visualised using KM467 labelled with Alexa Fluor 647. Images were obtained using a 40 × air objective on the Opera Phenix. Images were analysed in Harmony using predefined building blocks to segment nuclei and cytoplasm to define the number of cells, and to count individual bacteria (using Find Spots) within the cells. Macrophage phagocytosis (without complement) (**b**) and macrophage opsonophagocytosis (with complement) (**c**) was plotted as the average increase in bacterial uptake per cell in the presence of antibody. The average of 4 representative isolates per phenotype is shown and error bars represent standard deviation of 3 replicates. Significance was determined by ANOVA, Tukey's post-hoc (**b**) or Student’s t-test (**c**, * < 0.05, ** < 0.01, *** < 0.001).

To identify any potential functional differences between the antibody binding phenotypes, macrophage phagocytosis and opsonophagocytosis assays were carried out in the presence and absence of KM467. Assays were conducted in ultra-thin, flat bottom 96 well plates and imaged on the Opera Phenix using the 40 × air objective. Cells were stained with CellMask Orange (a plasma membrane stain), and DAPI (nuclear stain) and bacteria were visualised using KM467 labelled with Alexa Fluor 647. As the NB and WB isolates could not be visualised using KM467, serum from mice immunised with *E. coli* outer-membrane vesicles followed by an Alexa Fluor 647 secondary was used. Images were analysed in Harmony using predefined building blocks to segment nuclei and cytoplasm in the eukaryotic cells, to define the number of these cells, and to count individual bacteria within the cells (Fig. 5a, Supplementary Table S5). Initially, the macrophage phagocytosis assays were performed in the absence of complement. Although the bacterial cultures were OD600 adjusted to 1.00, there were significant differences in phagocytosis between isolates without antibody present (Supplementary Fig. S6). When plotting the average increase in phagocytosis in the presence of KM467, phagocytosis of the SB isolates increased by ~ 0.5 bacteria/cell while the SAB increased by ~ 3 bacteria/cell (Fig. 5b). No significant increase was observed for the WB and the NB isolates as expected. These results indicate that the KM467 agglutinated bacteria are more efficiently phagocytosed by macrophages.

The assay was then repeated for the SB and SAB isolates in the presence of baby rabbit complement (BRC) (Fig. 5c). The presence of complement and antibody augmented phagocytosis of the SAB isolates but not of the SB isolates, indicating that the antibody-induced agglutination may be more important than just antibody binding for efficient phagocytosis of *E. coli* ST131.

### Binding phenotypes correlate with differences in serum resistance and antibody-induced complement-dependent cytotoxicity (CDC)

**Figure 6:**
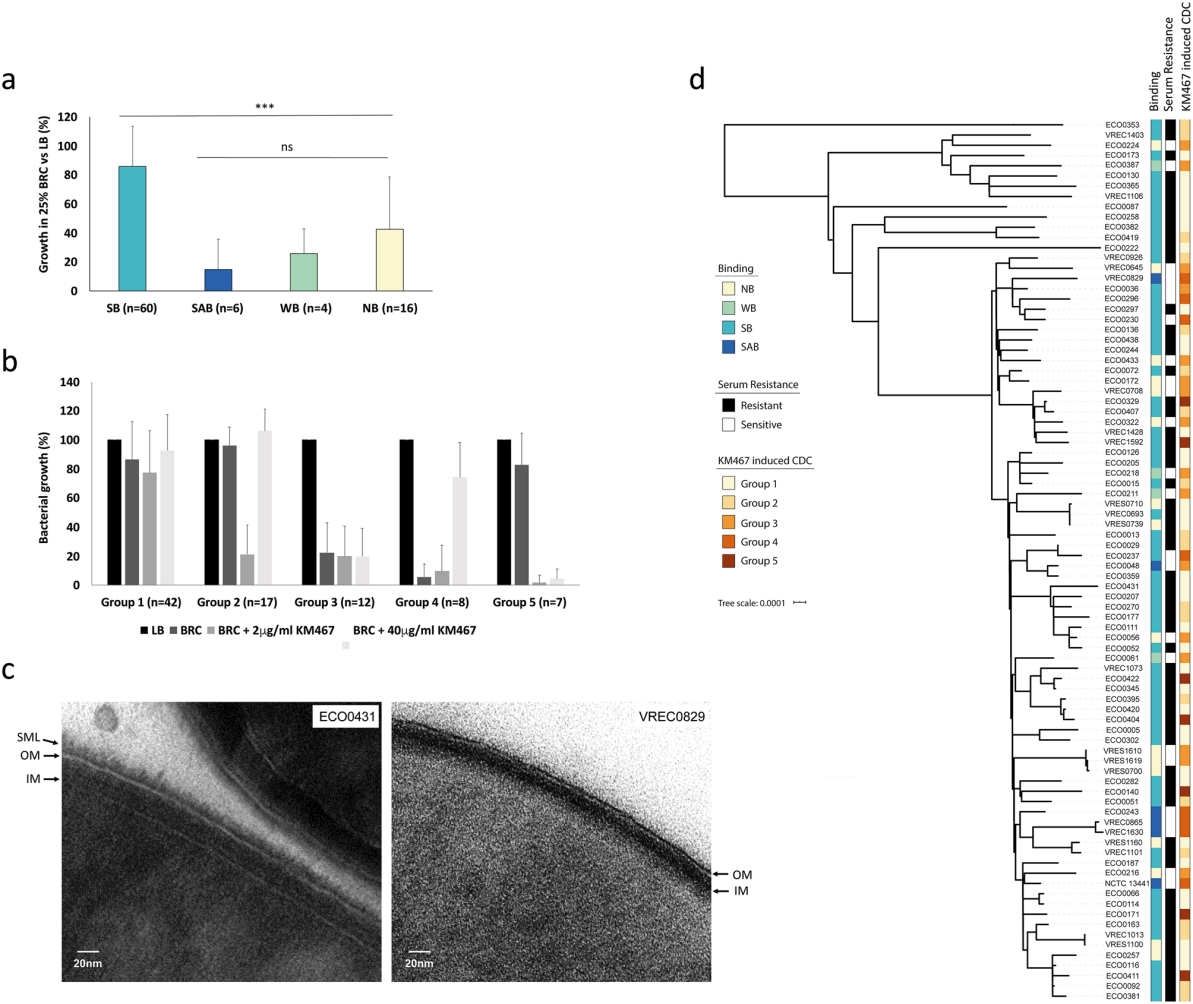
Differences in serum survival and complement-dependent cytotoxicity is linked to O-antigen density and correlate with binding phenotypes. Growth in LB broth in the presence or absence of 25% BRC was measured by OD600 after 4 h and the average for all isolates of each phenotype was plotted as percentage growth in the presence of serum compared to LB alone (**a**). Error bars represent standard deviation. Significance was determined by ANOVA, Tukey's post-hoc (* < 0.05, ** < 0.01, *** < 0.001). CDC was investigated for all isolates in the presence of 25% BRC alone, 25% BRC plus 2 or 40 µg/ml KM467 and plotted as percentage growth compared to LB alone (**b**). Growth patterns were grouped into 5 different CDC groups. Error bars represent standard deviation. A core genome maximum likelihood phylogenetic tree of the *E. coli* ST131 O25b isolates, created using RAxML v8.2.9 and visualised using iTOL version 5.5.1 (https://itol.embl.de), was aligned with the antibody binding phenotypes, serum resistance and antibody-induced CDC (**c**). Transmission electron microscopy images of an SAB (VREC0829) and an SB (ECO0431) isolate (**d**). Arrows indicate outer membrane (OM), inner membrane (IM), and surface matrix layer (SML).

To further evaluate the functional impact of the different antibody binding phenotypes, their serum resistance was evaluated using BRC. *E. coli* belonging to ST131 are the most commonly isolated *E. coli* from blood in bacteraemia patients, and have, not surprisingly, previously been characterised as being serum resistant^16^. Isolates that exhibited a SB phenotype generally grew well in serum (> 80% compared to LB) (Fig. 6a). However, the remaining isolates (that show either no, weak and agglutinating binding with the antibody) grew to different extents in serum. The WB and NB isolates showed ~ 30% and ~ 40% survival respectively, and the SAB isolates showed less than 20% survival compared to bacteria grown in LB (Fig. 6a). Comparable results were observed by using both optical density measurements (OD600) and colony forming unit (cfu) counting (Supplementary Fig. S7). Serum resistance did not seem to correlate with the source of the isolates nor with ESBL-plasmid carriage (Supplementary Table S1), which has previously been linked to enhanced serum resistance^30^.

To determine if KM467 could induce CDC in any of the isolates, survival in the presence of BRC and two different concentrations of KM467 was investigated. Interestingly, the isolates could be classified into five groups based on the CDC results (Fig. 6b). Group 1 (n = 42): were resistant to complement even in the presence of antibody. Group 2 (n = 17): were resistant to complement, but susceptible when 2 µg/ml KM467 was present, however, in the presence of increased amount of KM467 (40 µg/ml) they were protected from complement cytotoxicity. Group 3 (n = 12): were sensitive to complement alone and in both KM467 concentrations. Group 4 (n = 8): were susceptible to complement alone and in the presence of 2 µg/ml KM467, however, when 40 µg/ml KM467 was added these isolates were protected from complement cytotoxicity. Group 5 (n = 7): were complement resistant, but susceptible at both KM467 concentrations. These results were aligned to the phylogenetic tree of the isolates with the KM467 binding data (Fig. 6c). The majority of SB isolates were serum resistant, and all SAB and WB isolates were serum sensitive, whereas the NB phenotype did not correlate with serum resistance. The NB isolates that displayed either rough LPS or shorter O-antigen repeats by LPS gel electrophoresis were all serum sensitive. All isolates that have an agglutinating phenotype in the presence of KM467, except ECO0048, fell in CDC group 4, all WB isolates in group 3, and most SB isolates were in groups 1 and 2. In these experiments, the majority of isolates were resistant to complement even in the presence of the KM467, and a significant proportion of the remaining susceptible isolates were protected from complement killing in high antibody concentrations.

The finding that antibody binding phenotypes correlate with different levels of serum resistance might suggest different O-antigen densities. Transmission electron microscopy (TEM) was performed on an SB (group 1, serum and CDC resistant) and an SAB (group 4, serum sensitive) isolate. The TEM images revealed differences in the thickness of surface matrix surrounding their outer membrane, which is likely due to O-antigen density (Fig. 6d). The serum resistant SB isolate ECO0431 displayed a high amount of surface matrix (~ 14 nm), whereas the serum sensitive SAB isolate VREC0829 only had a low amount (~ 2 nm, Supplementary Fig. S8). This observation suggests that the surface matrix density is likely important for protection against serum killing in *E. coli* ST131 O25b isolates. Taken together these data suggest that an agglutinating binding phenotype may be linked to a less dense surface matrix layer and that this enhances both antibody-mediated phagocytosis and complement susceptibly.

## Discussion

HCI offers the opportunity to analyse bacterial populations at single cell resolution, which enables the visualisation and analysis of direct antibody binding to their individual bacterial targets at scale. This imaging can be undertaken at high-throughput, in 96-well microtiter plates, and the same analysis can be compared across multiple plates. Consequently, this approach has the potential to enable screening of a large number of candidate mAbs against a reference bacterial isolate, followed by functional screening of the most promising mAbs against large panels of clinically relevant isolates. HCI analysis software can carry out automated morphological image analysis which, for example, measures percentage binding, binding intensity and cell shape (single cell or agglutination).

Here, we have demonstrated that HCI can be used to screen a therapeutic mAb candidate against bacteria in high-resolution and in high-throughput, allowing us to study direct binding of mAbs to their native targets. When screening an O25b O-antigen mAb against a panel of 86 closely related clinical *E. coli* ST131 O25b:H4 isolates, distinct binding phenotypes were identified that correlated with mAb function both in CDC and phagocytosis assays. Despite all isolates being genetically typed as O25b, 16 of the 86 isolates showed no binding and four showed weak binding to the antibody. These binding phenotypes correlated with differences in O-antigen presentation compared to the isolates that showed strong antibody binding. For the majority of isolates (10/16 NB, 4/4 of WB) this could be tentatively explained by the presence of an IS*1* insertion sequence or mutations in genes in O-antigen or LPS core biosynthesis gene loci, which impacted the quantity, structure, or length of the repeating O-antigen units. Thus, it is possible to link genotype to phenotype, directing more intensive experimental investigations.

The majority (66/86) of isolates strongly bound the antibody, with six out of these displaying an agglutinating binding phenotype. Interestingly, the agglutinated isolates were more efficiently phagocytosed by macrophages in comparison to the non-agglutinated isolates and in addition, the agglutinating isolates were markedly more sensitive to complement killing. The association between the agglutinating phenotype with increased phagocytosis is not surprising as larger clusters of bacteria are more efficiently phagocytosed than individual bacteria; this has been observed previously for other bacterial species^31^. Agglutination may be linked with a less dense surface matrix layer (likely O-antigen) compared to the non-agglutinating SB isolates, although more work would be required to confirm this. O-antigen is known to protect against complement killing^32^ and O-antigen density has recently been directly linked to colicin protection in *E. coli* ST131^33^. In addition, complement sensitivity was observed in all NB and WB isolates that displayed either rough LPS or shorter O-antigen repeats.

It was evident that the phylogenetic position of individual isolates did not correlate with the binding phenotypes nor antibody function. This indicates that significant phenotypic variation can occur within highly clonal microbial populations. Similar data has been reported for other bacteria, including *Salmonella enterica* serovar Typhimurium^34^. However, the binding phenotypes correlated with both differences in phagocytosis and serum sensitivity, signifying the importance of having high-throughput methods to phenotypically screen large populations of isolates as genomic data alone cannot not always be predictive of a phenotype.

The majority of isolates screened in this study were resistant to complement, even in the presence of the KM467, and a significant proportion of the remaining susceptible isolates were protected from complement killing in the presence of increased KM467 concentrations. Antibody-mediated protection from complement has been observed previously^35–37^ and is a major concern when it comes to antibody therapy. This observation needs to be considered when targeting bacterial O-antigens by therapeutic mAbs. Our study also highlights the importance of screening candidate mAbs against a large panel of clinical isolates as there can be substantial phenotypic differences between genetically related organisms. Of note is that the NCTC13441 reference isolate is part of the minority SAB group and is perhaps not the best representative to be used for studies on *E. coli* ST131 O25b.

Compared to alternative mAb screening methods, HCI offers a much more comprehensive approach than ELISA or flow cytometry based assays. In terms of measuring antibody affinity, this is performed by measuring the intensity of the fluorescently-labelled antibody and does not provide any binding kinetic data (*K*_on_ or *K*_off_ values) that can be obtained by surface plasmon resonance. However, HCI can measure the binding of antibodies to native epitopes on intact bacteria, providing a more realistic in vivo representation. Phenotypic antibody screening, using an ELISA-based whole bacterial binding assay, was successfully used to discover a therapeutic mAb against the *Pseudomonas aeruginosa* exopolysaccharide Psl, a target which would have been missed using target-based screening methods^38^. A bispecific antibody MEDI3902, targeting Psl and PcrV (inhibiting type III secretion), recently completed phase 1 clinical trials^39,40^.

HCI can potentially be used to evaluate the binding efficacy and specificity of targeted mAbs against specific high-risk clones such as *E. coli* ST131, *Klebsiella pneumoniae* ST258 or *Staphylococcus aureus* ST22 (MRSA). This approach may be useful both in evaluating antibodies for their diagnostic potential, as well as for therapeutic use in combination with high-throughput opsonophagocytosis assays. It is also technically possible to screen different species simultaneously with the same antibody to evaluate cross-reactivity of broader spectrum targets including common surface antigens found on multiple pathogens. However, one of the potential advantages to more targeted mAbs is that these should not impact the host microbiota diversity, which is a major problem with antimicrobial usage.

Currently, HCI analysis software packages are optimised for mammalian cells and analysing bacteria this way has only just started being possible. We are hopeful that advances in image analysis and machine learning will soon enable more efficient segmentation and image analysis of bacteria. Our study was able to link phenotype with function, generating the possibility to be able to model the efficacy of a mAb based on the HCI phenotypic profile. Future application of HCI will likely not be limited to mAb screening, but also bispecific antibodies and antibody cocktails, as well as mAbs in combination with antimicrobials or novel antibacterial compounds.

## Materials and methods

### Bacterial isolates

The *E. coli* reference strain NCTC13441 was obtained from the Public Health England NCTC culture collection. Clinical *E. coli* ST131 (Supplementary Table S1) were isolated between 2007 and 2015 at Addenbrooke’s Hospital, Cambridge, UK, and whole genome sequence data was previously published^41^. Bacteria were cultured on LB agar plates and single colonies picked for overnight culture in LB broth at 37 °C at 200 rpm.

### Antibody

DNA encoding light chain variable region (DIVMTQTPLSLPVTPGEPASISCRSSQSLVHS DGNTYLHWYLQKPGQSPQLLIYTVSNRLSGVPDRFSGSGSGTDFTLKISRVEAEDVGVYYCSQSTHFPWTFGGGTKVEIK) and heavy chain variable region (QVQLVESGGGLV KPGGSLRLSCAASGFTFS DYYMYWIRQAPGKGLEWVSTISDGGTNIYYTDNVKGRF TISRDNAKNSLYLQMNSLRTEDTAVYFCARAAWWFAVDYWGQGTLVTVSS) amino acid sequences for 3E9-11 were synthesied and cloned into a plasmid vector for expression on a human IgG1 backbone. Vectors were transfected into Chinese Hamster Ovary cell lines for transient expression and purification. For the phagocytosis experiments, the antibody directly conjugated to Alexa Fluor 647 using an Alexa Fluor 647 Antibody Labelling Kit (Thermo Fisher).

### High-content antibody binding assay

Overnight bacterial cultures were diluted 1:200 in LB broth and 50 µl bacterial suspension was added per well in CellCarrier-96 Ultra plates (Perkin Elmer) and left in a static incubator at 37 °C for 2 h. The supernatant and any non-adhered bacteria were removed and the remaining bacteria were fixed with 4% paraformaldehyde (PFA) in phosphate buffered saline (PBS) for 15 min at room temperature, then washed with PBS. KM467 was diluted to 1 μg/ml in PBS + 1% bovine serum albumin (BSA) and added to the plate for 1 h (100 µl per well) at room temperature, after which it was replaced by 100 µl 2 μg/ml Alexa Fluor 647 Goat Anti-Human IgG (Thermo Fisher) plus 2 μg/ml DAPI (Thermo Fisher) in PBS + 1% BSA for 30 min in the dark. The plate was washed once with PBS, then 50 µl PBS was added to each well. The plates were imaged on an Opera Phenix using the Alexa Fluor 647 and DAPI channels and the 63 × water immersion objective and 16 fields and 3 Z-stacks were imaged per well.

### Opera Phenix image analysis

Image analysis was carried out using Perkin Elmer Harmony version 4.8. Supplementary Table 2 shows the detailed analysis workflow and outputs. Exported image data was plotted in R.

### LPS analysis

LPS was extracted using a modified hot-phenol method^42^. Ten ml bacterial overnight cultures were centrifuged for 10 min at 9,000 × g. The pellets were resuspended in 0.75 ml distilled water, then 0.5 ml 90% phenol was added. The mixture was vortexed then heated to 65 °C for 15 min, vortexing every 5 min. It was then cooled on ice after which the layers were separated by centrifugation at 9,000 × g for 20 min at 4 °C. The aqueous layer was dialysed against distilled water in 6,000–8,000 Dalton (Da) molecular weight cut off dialysis tubing (Spectra/Por, Spectrum) for 48 h, changing the water 5 times. 10 μl of each LPS was mixed with 2 × Novex Tris–Glycine SDS Sample Buffer and separated by gel electrophoresis for 1.5 h at 30 mA using Novex WedgeWell 12% Tris–Glycine Mini gels in Novex Tris–Glycine SDS Running Buffer (Thermo Fisher). The gels were stained using sodium-m-periodate silver staining as described previously^43^.

### Immunoblotting

Five μl of each LPS was separated by electrophoresis as described above. LPS was transferred to 0.2 μm polyvinylidene difluoride (PVDF) membranes using the Trans-Blot Turbo blotting system (Bio-Rad) with a high-molecular-weight (MW) program (1.3A up to 25 V for 10 min). The membranes were blocked overnight in 5% BSA in PBS plus 0.1% Tween 20 (PBST), then incubated with 1 μg/ml KM467 or polyclonal anti-O25 typing sera (Abcam, diluted 1:10) in 0.5% BSA in PBST for 2 h at RT. The membranes were washed 3 × 10 min in PBST, then incubated with 0.1 μg/ml HRP-conjugated Goat Anti-Human IgG H&L or HRP-conjugated Goat Anti-Rabbit IgG H&L (Abcam) in 0.5% BSA in PBST for 1 h at RT. The membranes were washed 6 × 10 min in PBST, then developed with SuperSignal West Pico PLUS (Thermo Scientific).

### Phylogenomic and SNP analysis

For SNP analysis, paired end reads from 86 *E. coli* ST131 O25b:H4 isolates were mapped to the reference sequence of *E. coli* strain Escherichia_coli_UPEC_ST131/NCTC13441 (accession number: GCA_900448475.1) using the RedDog mapping pipeline (V1beta.10.3), available at: https://github.com/katholt/reddog. For phylogenetic analyses, SNPs with confident homozygous allele calls (i.e. phred score > 20) outside of phage regions identified using PHAST^44^, and repetitive regions were identified using mummer (v3.23)^45^ (784,141 bp), and all genomes were concatenated to produce an alignment of alleles at 23,924 variant sites. Any further recombinant regions were identified using Gubbins (v2.3.2)^46^ and excluded resulting in a final set of 3,300 SNPs identified from an alignment of 5,174,631 bp for the 86 isolates. Maximum likelihood (ML) phylogenetic trees were inferred from this alignment using RAxML (v8.2.9)^47^, with a generalized time-reversible model, a Gamma distribution to model site-specific rate variation (the GTR + Γ substitution model; GTRGAMMA in RAxML), and 100 bootstrap pseudo-replicates to assess branch support. The resulting tree was visualized using iTOL version 5.5.1 (https://itol.embl.de)^48^.

SNPs in the Quinolone Resistance Determining Region (QRDR) of genes *gyrA, and parC* were extracted from the whole genome SNP alignments.

### Gene content and mobile genetic element analysis

SRST2 (v0.2.0)^22^ was used to identify AMR genes (ARGannot^49^), *E. coli* serotypes (EcOH database^23^), as well as their precise alleles. ISMapper^27^ was run with default parameters to screen all read sets for the presence of the transposases IS*1* (accession number: X52534.1) and IS*Ec10* (accession number: WP_000174402.1) relative to the NCTC13441 reference chromosome sequence.

Raw read data was assembled de novo with Unicycler (v0.4.7)^24^**,** visualised with Bandage (v0.8.1)^50^ and annotated with PROKKA (v1.13.3)^51^**,** and operon alignment figures generated using EasyFig2.2.2^52^. Individual genes as well as operons were extracted manually and analysed using NCBI BLAST, MAUVE^26^ and Clustal Omega^53^.

### Electron microscopy

Bacterial colonies were picked off LB agar plates and placed into planchettes with Hexedecene for cryopreservation in a Baltec HPM010 high pressure freezer. Frozen samples were post-fixed by freeze-substitution as described previously^54^. Ultrathin section were cut on a Leica UC6 ultramicrotome, contrasted with uranyl acetate and lead citrate and imaged on an FEI Spirit Biotwin TEM using a Tietz F416 CCD. The thickness of the surface matrix was quantified directly at 60 K magnification using EM Menu Measure at 20 locations from a total of 10 individual cells representing each isolate.

### Serum resistance

Bacterial overnights were diluted 1:1,000 in LB and 50 μl of each isolate was mixed with 25 μl of Dulbecco’s Phosphate-Buffered Saline with calcium and magnesium (DPBS + +) and 25 μl baby rabbit complement serum (BRC) (Bio-Rad, C12CA, lot 148,004). As controls, 50 μl of the diluted bacterial culture mixed with 25 μl DPBS++ and 25 μl LB was used. Plates were shaken at 200 rpm at 37 °C and OD600 values were obtained by an automated plate reader (BMG Fluostar) after 4 h. In parallel, tenfold serial dilutions were plated in triplicate and cfu were counted and the mean cfu/ml calculated.

### Antibody induced serum killing

Bacterial overnights were diluted 1:1,000 in LB. The sample was split into 3 tubes and KM467 was added to two tubes at final concentrations of 2 µg/ml or 40 µg/ml. The third sample was left as a control without antibody. These were incubated for 30 min shaking at 200 RPM at 37 °C, then 50 µl of these were mixed with 25 µl DPBS +  + and BRC, and assayed as described above.

### Phagocytosis and opsonophagocytosis assays

An average of 5 × 10^4^ THP1 cells per well were differentiated in CellCarrier-96 ultra microplates with 10 ng/ml phorbol-12-myristate-13 acetate (PMA) in RPMI + HEPES + heat inactivated foetal calf serum (HiFCS) (Labtech) for 3 days after which the media was changed to RPMI + HEPES + HiFCS. Overnight bacterial cultures were adjusted to an OD600 of 1 and diluted 1:100 in LB and pre-incubated with either KM467 (final concentration of 4 µg/ml) or LB (control). Bacteria were incubated for 1 h at 200 rpm at 37 °C, after which 50 µl was used to infect each well. Cells were infected for 90 min at 37 °C, after which the infection media was aspirated and extracellular bacteria were removed by 3 × 50 µl PBS washes. The plates were then fixed with 4% PFA. Macrophages were permeabilized with 0.1% Triton X-100 for 10 min followed blocking for 30 min with 10% BSA at room temperature in the dark. Bacteria were stained with 2 µg/ml Alexa Fluor 647 labelled KM467 antibody or serum from mice immunised with *E. coli* MG1655 outer-membrane vesicles (provided by Kymab Ltd) followed by an anti-mouse IgG Alexa Fluor 647 secondary (Abcam), diluted in 2% BSA for one hour in the dark. The cells were then stained with DAPI and CellMask Orange (Thermo Fisher) for 10 min in the dark. The plates were imaged on the Opera Phenix using the CellMask Orange, Alexa Fluor 647 and DAPI channels and the 40 × air objective, and 11 fields and 3 Z-stacks were imaged per well.

Opsonophagocytosis assays were performed in a same way as the phagocytosis assays, except the dilution of the overnights was done in LB with 10% BRC.

### Statistical analysis

Statistical analysis was carried using Student's t-test or one-way ANOVA analysis of variance, followed by post hoc pairwise comparison using Tukey’s tests.

## Supplementary information

Supplementary Table S4.

Supplementary Information.

Supplementary Table S1.

Supplementary Table S3.

## Data Availability

Whole genome sequenced raw read data is available at the European Nucleotide Archive (ENA) and individual sample accession numbers are listed in Supplementary Table S1. The image data analysed in this study can be found in Supplementary Table S3, and images generated during this study are available from the corresponding author on reasonable request.
