# Publisher Correction: A novel therapeutic antibody screening method using bacterial high-content imaging reveals functional antibody binding phenotypes of *Escherichia coli* ST131

**DOI:** 10.1038/s41598-021-86902-y

**Published:** 2021-03-26

**Authors:** Mailis Maes, Zoe A. Dyson, Sarah E. Smith, David A. Goulding, Catherine Ludden, Stephen Baker, Paul Kellam, Stephen T. Reece, Gordon Dougan, Josefin Bartholdson Scott

**Affiliations:** 1 Department of Medicine, Cambridge Institute for Therapeutic Immunology & Infectious Disease, University of Cambridge, Cambridge, UK; 2 Department of Infectious Diseases, Central Clinical School, Monash University, Melbourne, VIC 3004 Australia; 3 London School of Hygiene and Tropical Medicine, London, UK; 4 Kymab Ltd, Babraham Research Campus, Cambridge, UK; 5 Wellcome Sanger Institute, Hinxton, UK; 6 Department of Infectious Disease, Imperial College London, London, UK

Correction to: *Scientific Reports* https://doi.org/10.1038/s41598-020-69300-8, published online 24 July 2020

In the original version of this Article, the author Josefin Bartholdson Scott was incorrectly indexed. This error has now been corrected.

