# Supplementary material for: A novel therapeutic antibody screening method using bacterial high-content imaging reveals functional antibody binding phenotypes of Escherichia coli ST131
Source: Sci Rep. 2020 Jul 24;10:12414. doi: 10.1038/s41598-020-69300-8 (PMC7382476; doi:10.1038/s41598-020-69300-8)
Supplement: Supplementary file 2 — Supplementary Information. [file 41598_2020_69300_MOESM2_ESM.pdf]

# A novel therapeutic antibody screening method using bacterial high-content imaging reveals functional antibody binding phenotypes of *Escherichia coli* ST131

Mailis Maes, Zoe A. Dyson, Sarah E. Smith, David A. Goulding, Catherine Ludden, Stephen Baker, Paul Kellam, Stephen T. Reece, Gordon Dougan, Josefin Bartholdson Scott

## Supplementary Method

*Direct TMB ELISA*

High bind ELISA plates (Costar) were coated with 50µL of LPS from *E. coli* NCTC13441 or control LPS from *E. coli* O111:B4 (Sigma) in duplicate at an estimated 30µg/mL, 20µg/mL, 10µg/mL or 2.5 µg/mL in phosphate buffered saline (PBS) overnight at 4°C. Plates were washed 3x with PBS+0.1% Tween (PBST), blocked with PBS+1% bovine serum albumin (BSA) for 1h at RT, then washed again 3x with PBST. Serial 3-fold dilutions of KM467 (50µg/ml starting concentration) were prepared in PBS+1% BSA and 50ul per well was added to the coated wells and incubated for 1h at RT. Plates was washed 3x with PBST, after which 50µL of horseradish peroxidase (HRP) conjugated goat anti-human IgG diluted 1:3000 in PBS+1% BSA was added for 1 hr at RT. Plates was washed 3x with PBST, then 50µl of TMB substrate (Sigma) was added and incubated at RT protected from light. Then 50µl/well of stop solution (sulphuric acid, Fisher) was added and absorbance was read at 450nm (and corrected at 540nm).

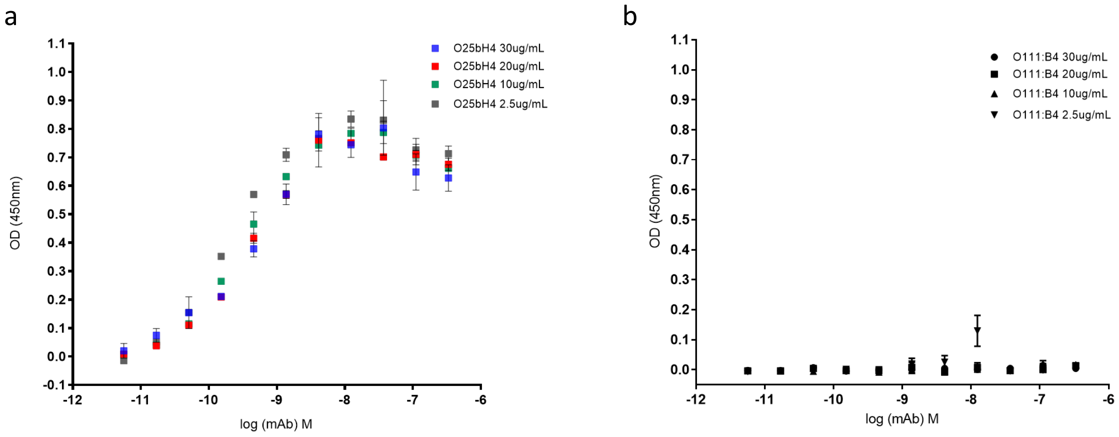


**Supplementary Figure S1: Titration of KM467 against *E. coli* ST131 NCTC13441 LPS by ELISA.** ELISA plates were coated with different amounts of isolated LPS from *E. coli* ST131 NCTC13441 O25b:H4 (**a**) and control LPS from *E. coli* O111:B4 (**b**), and serial 3-fold dilutions of KM467 (top concentration 50µg/ml) was added and binding was measured using TMB ELISA substrate at 405nm. Error bars represent standard deviation of 3 replicates.

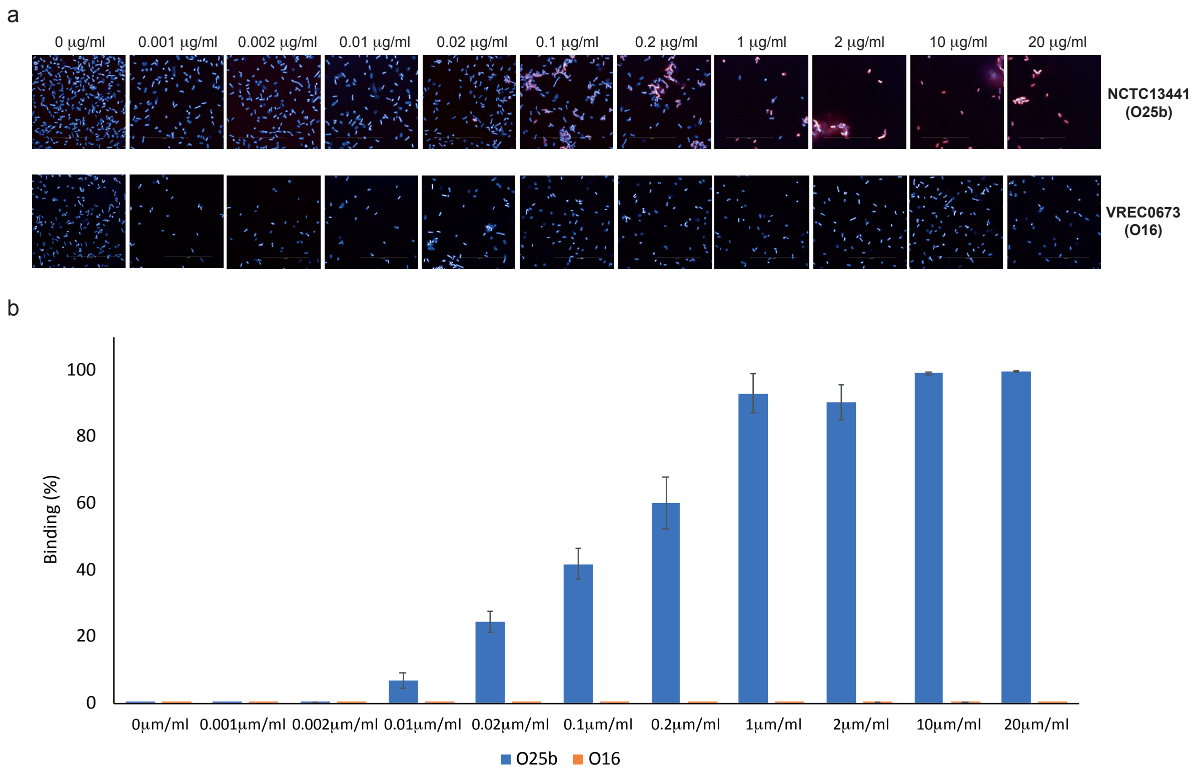


**Supplementary Figure S2: Antibody binding titration.** *E. coli* ST131 O25b reference isolate NCTC13441 and an O16 clinical isolate (VREC0673) were incubated with different concentrations of KM467 (ranging from 0-20μg/ml). Bacteria were stained with DAPI and an Alexa Fluor 647-conjugated secondary antibody (**a**). Percentage binding was plotted against antibody concentration (**b**). Error bars represent standard deviation of 4 replicates.

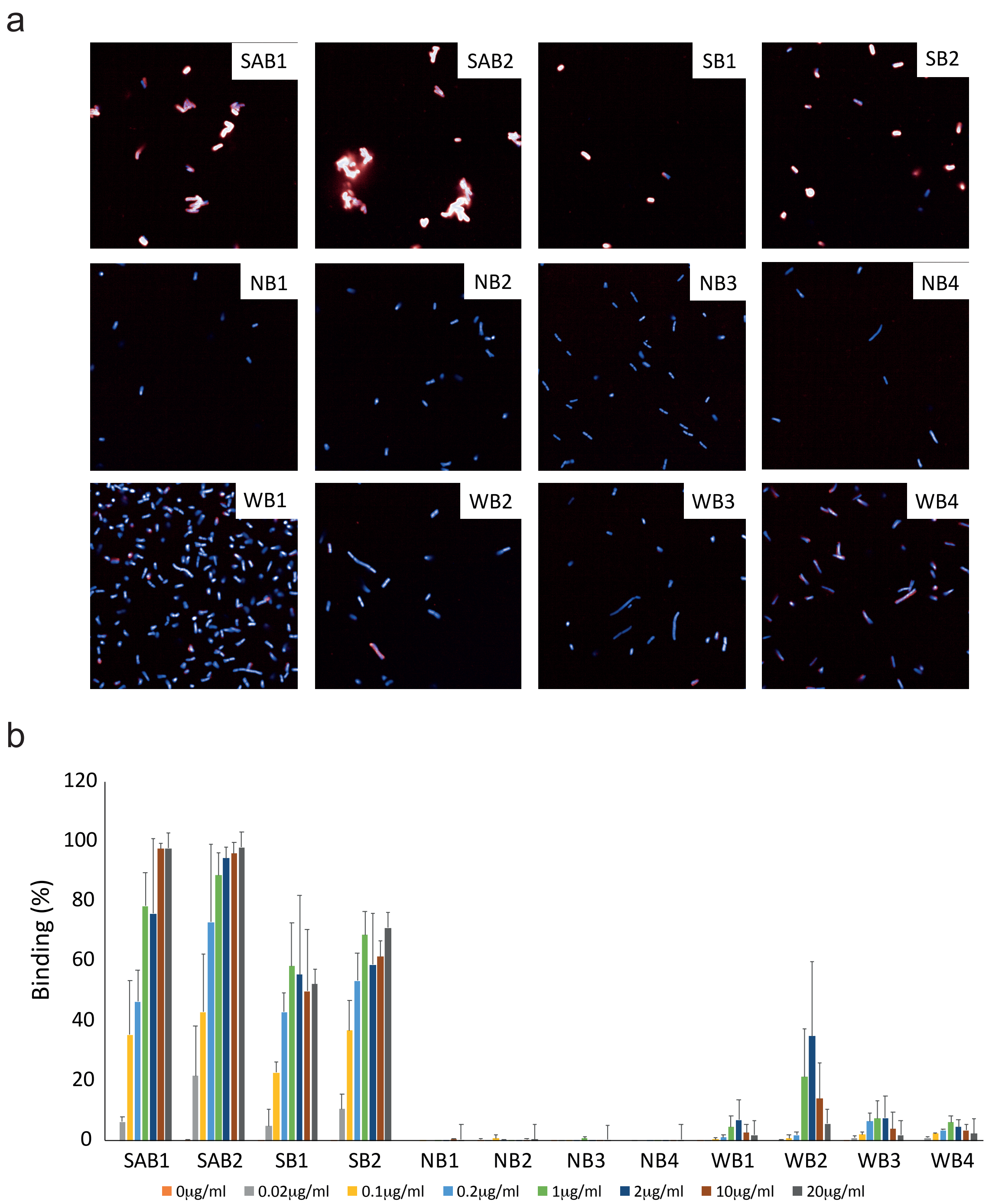


**Supplementary Figure S3: Antibody binding titration against different phenotypes confirms weak binding.** Representative isolates exhibiting different binding phenotype were incubated with different concentrations of KM467 (ranging from 0-20μg/ml). Bacteria were stained with DAPI and an Alexa Fluor 647-conjugated secondary antibody. Images taken at 1μg/ml are shown (**a**). Percentage binding was plotted against concentration (**b**). Error bars represent standard deviation of 3 replicates.

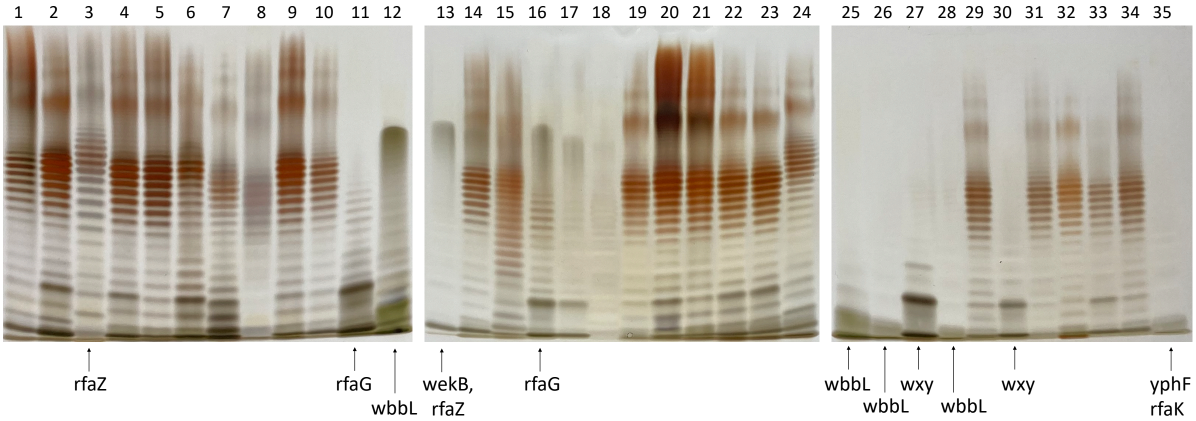


**Supplementary Figure S4: Silver stained LPS gels**. Isolated LPS from 35 isolates was analysed by gel electrophoresis followed by silver staining. Order on gel (1-35): VREC1101, VREC1106, VRES1160, VREC1403, VREC1428, VREC1592, VRES1610, VRES1619, VREC1630, NCTC13441, ECO0056, ECO0061, VREC0645, VREC0693, VRES0700, VREC0708, VRES0710, VRES0739, VREC0829, VREC0865, VREC0926, VREC1013, VREC1073, VRES1100, ECO0172, ECO0211, ECO0218, ECO0224, ECO0353, ECO0387, ECO0395, ECO0407, ECO0422, ECO0431, ECO0433. Any identified disrupted genes are indicated below gels.

**Supplementary Figure 5: Wzy, WekB, WbbL, RfaJ, RfaZ Clustal Omega (version 1.2.4) amino acid alignments** (https://www.ebi.ac.uk/Tools/msa/clustalo).

```
CLUSTAL O(1.2.4) multiple sequence alignment WbbL


ECO0211         MVYIIIVSHGHDDYIENLLLNLKLPSGRFKIIVRDNKSSMVLKKTCEKNCVTYLHGGQYE  60
NCTC13441       MVYIIIVSHGHDDYIENLLLNLKLPSGRFKIIVRDNKSSMVLKKTCEKNCVTYLHGGQYG  60
VREC1403        MVYIIIVSHGHDDYIENLLLNLKLPSGRFKIIVRDNKSSMVLKKTCEKNCVTYLHGGQYG  60
ECO0218         MVYIIIVSHGHDDYIENLLLNLKLPSGRFKIIVRDNKSSMVLKKTCEKNCVTYLHGGQYG  60
ECO0061         MVYIIIVSHGHDDYIENLLLNLKLPSGRFKIIVRDNKSSMVLKKTCEKNCVTYLHGGQYG  60
ECO0387         MVYIIIVSHGHDDYIENLLLNLKLPSGRFKIIVRDNKSSMVLKKTCEKNCVTYLHGGQYG  60
VRES1610        MVYIIIVSHGHDDYIENLLLNLKLPSGRFKIIVRDNKSSMVLKKTCEKNCVTYLHGGQYG  60
VRES1619        MVYIIIVSHGHDDYIENLLLNLKLPSGRFKIIVRDNKSSMVLKKTCEKNCVTYLHGGQYG  60
VRES0739        MVYIIIVSHGHDDYIENLLLNLKLPSGRFKIIVRDNKSSMVLKKTCEKNCVTYLHGGQYG  60
VREC0645        MVYIIIVSHGHDDYIENLLLNLKLPSGRFKIIVRDNKSSMVLKKTCEKNCVTYLHGGQYG  60
VRES1160        MVYIIIVSHGHDDYIENLLLNLKLPSGRFKIIVRDNKSSMVLKKTCEKNCVTYLHGGQYG  60
VRES0700        MVYIIIVSHGHDDYIENLLLNLKLPSGRFKIIVRDNKSSMVLKKTCEKNCVTYLHGGQYG  60
VREC1630        MVYIIIVSHGHDDYIENLLLNLKLPSGRFKIIVRDNKSSMVLKKTCEKNCVTYLHGGQYG  60
VREC1428        MVYIIIVSHGHDDYIENLLLNLKLPSGRFKIIVRDNKSSMVLKKTCEKNCVTYLHGGQYG  60
VREC1106        MVYIIIVSHGHDDYIENLLLNLKLPSGRFKIIVRDNKSSMVLKKTCEKNCVTYLHGGQYG  60
VREC1101        MVYIIIVSHGHDDYIENLLLNLKLPSGRFKIIVRDNKSSMVLKKTCEKNCVTYLHGGQYG  60
VREC1073        MVYIIIVSHGHDDYIENLLLNLKLPSGRFKIIVRDNKSSMVLKKTCEKNCVTYLHGGQYG  60
VREC1013        MVYIIIVSHGHDDYIENLLLNLKLPSGRFKIIVRDNKSSMVLKKTCEKNCVTYLHGGQYG  60
VREC0926        MVYIIIVSHGHDDYIENLLLNLKLPSGRFKIIVRDNKSSMVLKKTCEKNCVTYLHGGQYG  60
VREC0865        MVYIIIVSHGHDDYIENLLLNLKLPSGRFKIIVRDNKSSMVLKKTCEKNCVTYLHGGQYG  60
VREC0829        MVYIIIVSHGHDDYIENLLLNLKLPSGRFKIIVRDNKSSMVLKKTCEKNCVTYLHGGQYG  60
VREC0693        MVYIIIVSHGHDDYIENLLLNLKLPSGRFKIIVRDNKSSMVLKKTCEKNCVTYLHGGQYG  60
ECO0433         MVYIIIVSHGHDDYIENLLLNLKLPSGRFKIIVRDNKSSMVLKKTCEKNCVTYLHGGQYG  60
ECO0224         MVYIIIVSHGHDDYIENLLLNLKLPSGRFKIIVRDNKSSMVLKKTCEKNCVTYLHGGQYG  60
ECO0411         MVYIIIVSHGHDDYIENLLLNLKLPSGRFKIIVRDNKSSMVLKKTCEKNCVTYLHGGQYG  60
ECO0431         MVYIIIVSHGHDDYIENLLLNLKLPSGRFKIIVRDNKSSMVLKKTCEKNCVTYLHGGQYG  60
ECO0353         MVYIIIVSHGHDDYIENLLLNLKLPSGRFKIIVRDNKSSMVLKKTCEKNCVTYLHGGQYG  60
                *********************************************************** 

ECO0211         FGHNNNIAVSYIINNFMIMNNDYFLFLNPDVFITSESLINYVDYIISNDYKFSTLCLYRD  120
NCTC13441       FGHNNNIAVSYIINNFMIMNNDYFLFLNPDVFITSESLINYVDYIISNDYKFSTLCLYRD  120
VREC1403        FGHNNNIAVSYIINNFMIMNNDYFLFLNPDVFITSESLINYVDYIISNDYKFSTLCLYRD  120
ECO0218         FGHNNNIAVSYIINNFMIMNNDYFLFLNPDVFITSESLINYVDYIISNDYKFSTLCLYRD  120
ECO0061         FGHNNNIAVSYIINNFMIMNNDYFLFLNPDVFITSESLINYVDYIISNDYKFSTLCLYRD  120
ECO0387         FGHNNNIAVSYIINNFMIMNNDYFLFLNPDVFITSESLINYVDYIISNDYKFSTLCLYRD  120
VRES1610        FGHNNNIAVSYIINNFMIMNNDYFLFLNPDVFITSESLINYVDYIISNDYKFSTLCLYRD  120
VRES1619        FGHNNNIAVSYIINNFMIMNNDYFLFLNPDVFITSESLINYVDYIISNDYKFSTLCLYRD  120
VRES0739        FGHNNNIAVSYIINNFMIMNNDYFLFLNPDVFITSESLINYVDYIISNDYKFSTLCLYRD  120
VREC0645        FGHNNNIAVSYIINNFMIMNNDYFLFLNPDVFITSESLINYVDYIISNDYKFSTLCLYRD  120
VRES1160        FGHNNNIAVSYIINNFMIMNNDYFLFLNPDVFITSESLINYVDYIISNDYKFSTLCLYRD  120
VRES0700        FGHNNNIAVSYIINNFMIMNNDYFLFLNPDVFITSESLINYVDYIISNDYKFSTLCLYRD  120
VREC1630        FGHNNNIAVSYIINNFMIMNNDYFLFLNPDVFITSESLINYVDYIISNDYKFSTLCLYRD  120
VREC1428        FGHNNNIAVSYIINNFMIMNNDYFLFLNPDVFITSESLINYVDYIISNDYKFSTLCLYRD  120
VREC1106        FGHNNNIAVSYIINNFMIMNNDYFLFLNPDVFITSESLINYVDYIISNDYKFSTLCLYRD  120
VREC1101        FGHNNNIAVSYIINNFMIMNNDYFLFLNPDVFITSESLINYVDYIISNDYKFSTLCLYRD  120
VREC1073        FGHNNNIAVSYIINNFMIMNNDYFLFLNPDVFITSESLINYVDYIISNDYKFSTLCLYRD  120
VREC1013        FGHNNNIAVSYIINNFMIMNNDYFLFLNPDVFITSESLINYVDYIISNDYKFSTLCLYRD  120
VREC0926        FGHNNNIAVSYIINNFMIMNNDYFLFLNPDVFITSESLINYVDYIISNDYKFSTLCLYRD  120
VREC0865        FGHNNNIAVSYIINNFMIMNNDYFLFLNPDVFITSESLINYVDYIISNDYKFSTLCLYRD  120
VREC0829        FGHNNNIAVSYIINNFMIMNNDYFLFLNPDVFITSESLINYVDYIISNDYKFSTLCLYRD  120
VREC0693        FGHNNNIAVSYIINNFMIMNNDYFLFLNPDVFITSESLINYVDYIISNDYKFSTLCLYRD  120
ECO0433         FGHNNNIAVSYIINNFMIMNNDYFLFLNPDVFITSESLINYVDYIISNDYKFSTLCLYRD  120
ECO0224         FGHNNNIAVSYIINNFMIMNNDYFLFLNPDVFITSESLINYVDYIISNDYKFSTLCLYRD  120
ECO0411         FGHNNNIAVSYIINNFMIMNNDYFLFLNPDVFITSESLINYVDYIISNDYKFSTLCLYRD  120
ECO0431         FGHNNNIAVSYIINNFMIMNNDYFLFLNPDVFITSESLINYVDYIISNDYKFSTLCLYRD  120
ECO0353         FGHNNNIAVSYIINNFMIMNNDYFLFLNPDVFITSESLINYVDYIISNDYKFSTLCLYRD  120
                ************************************************************

ECO0211         FTKSKHDYSIRSFPTLYDFLCSFLLGVNKSKIKKENILSDTVVDWCAGSFMLIHALSFLN  180
NCTC13441       FTKSKHDYSIRSFPTLYDFLCSFLLGVNKSKIKKENILSDTVVDWCAGSFMLIHALSFLN  180
VREC1403        FTKSKHDYSIRSFPTLYDFLCSFLLGVNKSKIKKENILSDTVVDWCAGSFMLIHALSFLN  180
ECO0218         FTKSKHDYSIRSFPTLYDFLCSFLLGVNKSKIKKENILSDTVVDWCAGSFMLIHALSFLN  180
ECO0061         FTKSKHDYSIRSFPTLYDFLCSFLLGVNKSKIKKENILSDTVVDWCAGSFMLIHALSFLN  180
ECO0387         FTKSKHDYSIRSFPTLYDFLCSFLLGVNKSKIKKENILSDTVVDWCAGSFMLIHALSFLN  180
VRES1610        FTKSKHDYSIRSFPTLYDFLCSFLLGVNKSKIKKENILSDTVVDWCAGSFMLIHALSFLN  180
VRES1619        FTKSKHDYSIRSFPTLYDFLCSFLLGVNKSKIKKENILSDTVVDWCAGSFMLIHALSFLN  180
VRES0739        FTKSKHDYSIRSFPTLYDFLCSFLLGVNKSKIKKENILSDTVVDWCAGSFMLIHALSFLN  180
```

```
VREC0645       FTKSKHDYSIRSFPTLYDFLCSFLLGVNKSKIKKENILSDTVVDWCAGSFMLIHALSFLN  180
VRES1160       FTKSKHDYSIRSFPTLYDFLCSFLLGVNKSKIKKENILSDTVVDWCAGSFMLIHALSFLN  180
VRES0700       FTKSKHDYSIRSFPTLYDFLCSFLLGVNKSKIKKENILSDTVVDWCAGSFMLIHALSFLN  180
VREC1630       FTKSKHDYSIRSFPTLYDFLCSFLLGVNKSKIKKENILSDTVVDWCAGSFMLIHALSFLN  180
VREC1428       FTKSKHDYSIRSFPTLYDFLCSFLLGVNKSKIKKENILSDTVVDWCAGSFMLIHALSFLN  180
VREC1106       FTKSKHDYSIRSFPTLYDFLCSFLLGVNKSKIKKENILSDTVVDWCAGSFMLIHALSFLN  180
VREC1101       FTKSKHDYSIRSFPTLYDFLCSFLLGVNKSKIKKENILSDTVVDWCAGSFMLIHALSFLN  180
VREC1073       FTKSKHDYSIRSFPTLYDFLCSFLLGVNKSKIKKENILSDTVVDWCAGSFMLIHALSFLN  180
VREC1013       FTKSKHDYSIRSFPTLYDFLCSFLLGVNKSKIKKENILSDTVVDWCAGSFMLIHALSFLN  180
VREC0926       FTKSKHDYSIRSFPTLYDFLCSFLLGVNKSKIKKENILSDTVVDWCAGSFMLIHALSFLN  180
VREC0865       FTKSKHDYSIRSFPTLYDFLCSFLLGVNKSKIKKENILSDTVVDWCAGSFMLIHALSFLN  180
VREC0829       FTKSKHDYSIRSFPTLYDFLCSFLLGVNKSKIKKENILSDTVVDWCAGSFMLIHALSFLN  180
VREC0693       FTKSKHDYSIRSFPTLYDFLCSFLLGVNKSKIKKENILSDTVVDWCAGSFMLIHALSFLN  180
ECO0433        FTKSKHDYSIRSFPTLYDFLCSFLLGVNKSKIKKENILSDTVVDWCAGSFMLIHALSFLN  180
ECO0224        FTKSKHDYSIRSFPTLYDFLCSFLLGVNKSKIKKENILSDTVVDWCAGSFMLIHALSFLN  180
ECO0411        FTKSKHDYSIRSFPTLYDFLCSFLLGVNKSKIKKENILSDTVVDWCAGSFMLIHALSFLN  180
ECO0431        FTKSKHDYSIRSFPTLYDFLCSFLLGVNKSKIKKENILSDTVVDWCAGSFMLIHALSFLN  180
ECO0353        FTKSKHDYSIRSFPTLYDFLCSFLLGVNKSKIKKENILSDTVVDWCAGSFMLIHALSFLN  180
               ************************************************************

ECO0211        VNGFDQKYFMYCEDIDLCMRLKLSGVDLYYTPHFDAIHYAQHENRRIFTKAFRWHIRSIT  240
NCTC13441      VNGFDQKYFMYCEDIDLCMRLKLSGVDLYYTPHFDAIHYAQHENRRIFTKAFRWHIRSIT  240
VREC1403       VNGFDQKYFMYCEDIDLCMRLKLSGVDLYYTPHFDAIHYAQHENRRIFTKAFRWHIRSIT  240
ECO0218        VNGFDQKYFMYCEDIDLCMRLKLSGVDLYYTPHFDAIHYAQHENRRIFTKAFRWHIRSIT  240
ECO0061        VNGFDQKYFMYCEDIDLCMRLKLSGVDLYYTPHFDAIHYAQHENRRIFTKAF--------  232
ECO0387        VNGFDQKYFMYCEDIDLCMRLKLSGVDLYYTPHFDAIHYAQHENRRIFTKAFRWHIRSIT  240
VRES1610       VNGFDQKYFMYCEDIDLCMRLKLSGVDLYYTPHFDAIHYAQHENRRIFTKAFRWHIRSIT  240
VRES1619       VNGFDQKYFMYCEDIDLCMRLKLSGVDLYYTPHFDAIHYAQHENRRIFTKAFRWHIRSIT  240
VRES0739       VNGFDQKYFMYCEDIDLCMRLKLSGVDLYYTPHFDAIHYAQHENRRIFTKAFRWHIRSIT  240
VREC0645       VNGFDQKYFMYCEDIDLCMRLKLSGVDLYYTPHFDAIHYAQHENRRIFTKAFRWHIRSIT  240
VRES1160       VNGFDQKYFMYCEDIDLCMRLKLSGVDLYYTPHFDAIHYAQHENRRIFTKAFRWHIRSIT  240
VRES0700       VNGFDQKYFMYCEDIDLCMRLKLSGVDLYYTPHFDAIHYAQHENRRIFTKAFRWHIRSIT  240
VREC1630       VNGFDQKYFMYCEDIDLCMRLKLSGVDLYYTPHFDAIHYAQHENRRIFTKAFRWHIRSIT  240
VREC1428       VNGFDQKYFMYCEDIDLCMRLKLSGVDLYYTPHFDAIHYAQHENRRIFTKAFRWHIRSIT  240
VREC1106       VNGFDQKYFMYCEDIDLCMRLKLSGVDLYYTPHFDAIHYAQHENRRIFTKAFRWHIRSIT  240
VREC1101       VNGFDQKYFMYCEDIDLCMRLKLSGVDLYYTPHFDAIHYAQHENRRIFTKAFRWHIRSIT  240
VREC1073       VNGFDQKYFMYCEDIDLCMRLKLSGVDLYYTPHFDAIHYAQHENRRIFTKAFRWHIRSIT  240
VREC1013       VNGFDQKYFMYCEDIDLCMRLKLSGVDLYYTPHFDAIHYAQHENRRIFTKAFRWHIRSIT  240
VREC0926       VNGFDQKYFMYCEDIDLCMRLKLSGVDLYYTPHFDAIHYAQHENRRIFTKAFRWHIRSIT  240
VREC0865       VNGFDQKYFMYCEDIDLCMRLKLSGVDLYYTPHFDAIHYAQHENRRIFTKAFRWHIRSIT  240
VREC0829       VNGFDQKYFMYCEDIDLCMRLKLSGVDLYYTPHFDAIHYAQHENRRIFTKAFRWHIRSIT  240
VREC0693       VNGFDQKYFMYCEDIDLCMRLKLSGVDLYYTPHFDAIHYAQHENRRIFTKAFRWHIRSIT  240
ECO0433        VNGFDQKYFMYCEDIDLCMRLKLSGVDLYYTPHFDAIHYAQHENRRIFTKAFRWHIRSIT  240
ECO0224        VNGFDQKYFMYCEDIDLCMRLKLSGVDLYYTPHFDAIHYAQHENRRIFTKAFR-------  233
ECO0411        VNGFDQKYFMYCEDIDLCMRLKLSGVDLYYTPHFDAIHYAQHENRRIFTKAFRWHIRSIT  240
ECO0431        VNGFDQKYFMYCEDIDLCMRLKLSGVDLYYTPHFDAIHYAQHENRRIFTKAFRWHIRSIT  240
ECO0353        VNGFDQKYFMYCEDIDLCMRLKLSGVDLYYTPHFDAIHYAQHENRRIFTKAFRWHIRSIT  240
               ****************************************************

ECO0211        RYILRKPILSYKNYRKITSELVK263
NCTC13441      RYILRKPILSYKNYRKITSELVK263
VREC1403       RYILRKPILSYKNYRKITSELVK263
ECO0218        RYILRKPILSYKNYRKITSELVK263
ECO0061        -----------------------232
ECO0387        RYILRKPILSYKNYRKITSELVK263
VRES1610       RYILRKPILSYKNYRKITSELVK263
VRES1619       RYILRKPILSYKNYRKITSELVK263
VRES0739       RYILRKPILSYKNYRKITSELVK263
VREC0645       RYILRKPILSYKNYRKITSELVK263
VRES1160       RYILRKPILSYKNYRKITSELVK263
VRES0700       RYILRKPILSYKNYRKITSELVK263
VREC1630       RYILRKPILSYKNYRKITSELVK263
VREC1428       RYILRKPILSYKNYRKITSELVK263
VREC1106       RYILRKPILSYKNYRKITSELVK263
VREC1101       RYILRKPILSYKNYRKITSELVK263
VREC1073       RYILRKPILSYKNYRKITSELVK263
VREC1013       RYILRKPILSYKNYRKITSELVK263
VREC0926       RYILRKPILSYKNYRKITSELVK263
VREC0865       RYILRKPILSYKNYRKITSELVK263
VREC0829       RYILRKPILSYKNYRKITSELVK263
VREC0693       RYILRKPILSYKNYRKITSELVK263
ECO0433        RYILRKPILSYKNYRKITSELVK263
ECO0224        -----------------------233
ECO0411        RYILRKPILSYKNYRKITSELVK263
ECO0431        RYILRKPILSYKNYRKITSELVK263
ECO0353        RYILRKPILSYKNYRKITSELVK263
```

CLUSTAL O(1.2.4) multiple sequence alignment - **WekB**

```
NCTC13441      MKVAFLSAYDPLSTSSWSGTPYYMLKALSKRNISIEILGPVNSYMIYMLKVYKLILRCFG  60
VREC1403       MKVAFLSAYDPLSTSSWSGTPYYMLKALSKRNISIEILGPVNSYMIYMLKVYKLILRCFG  60
ECO0218        MKVAFLSAYDPLSTSSWSGTPYYMLKALSKRNISIEILGPVNSYMIYMLKVYKLILRCFG  60
ECO0061        MKVAFLSAYDPLSTSSWSGTPYYMLKALSKRNISIEILGPVNSYMIYMLKVYKLILRCFG  60
ECO0211        MKVAFLSAYDPLSTSSWSGTPYYMLKALSKRNISIEILGPVNSYMIYMLKVYKLILRCFG  60
ECO0387        MKVAFLSAYDPLSTSSWSGTPYYMLKALSKRNISIEILGPVNSYMIYMLKVYKLILRCFG  60
VRES1610       MKVAFLSAYDPLSTSSWSGTPYYMLKALSKRNISIEILGPVNSYMIYMLKVYKLILRCFG  60
VRES1619       MKVAFLSAYDPLSTSSWSGTPYYMLKALSKRNISIEILGPVNSYMIYMLKVYKLILRCFG  60
VRES0739       MKVAFLSAYDPLSTSSWSGTPYYMLKALSKRNISIEILGPVNSYMIYMLKVYKLILRCFG  60
VREC0645       MKVAFLSAYDPLSTSSWSGTPYYMLKALSKRNISIEILGPVNSYMIYMLKVYKLILRCFG  60
VRES1160       MKVAFLSAYDPLSTSSWSGTPYYMLKALSKRNISIEILGPVNSYMIYMLKVYKLILRCFG  60
VRES0700       MKVAFLSAYDPLSTSSWSGTPYYMLKALSKRNISIEILGPVNSYMIYMLKVYKLILRCFG  60
VREC1630       MKVAFLSAYDPLSTSSWSGTPYYMLKALSKRNISIEILGPVNSYMIYMLKVYKLILRCFG  60
VREC1428       MKVAFLSAYDPLSTSSWSGTPYYMLKALSKRNISIEILGPVNSYMIYMLKVYKLILRCFG  60
VREC1106       MKVAFLSAYDPLSTSSWSGTPYYMLKALSKRNISIEILGPVNSYMIYMLKVYKLILRCFG  60
VREC1101       MKVAFLSAYDPLSTSSWSGTPYYMLKALSKRNISIEILGPVNSYMIYMLKVYKLILRCFG  60
VREC1073       MKVAFLSAYDPLSTSSWSGTPYYMLKALSKRNISIEILGPVNSYMIYMLKVYKLILRCFG  60
VREC1013       MKVAFLSAYDPLSTSSWSGTPYYMLKALSKRNISIEILGPVNSYMIYMLKVYKLILRCFG  60
VREC0926       MKVAFLSAYDPLSTSSWSGTPYYMLKALSKRNISIEILGPVNSYMIYMLKVYKLILRCFG  60
VREC0865       MKVAFLSAYDPLSTSSWSGTPYYMLKALSKRNISIEILGPVNSYMIYMLKVYKLILRCFG  60
VREC0829       MKVAFLSAYDPLSTSSWSGTPYYMLKALSKRNISIEILGPVNSYMIYMLKVYKLILRCFG  60
VREC0693       MKVAFLSAYDPLSTSSWSGTPYYMLKALSKRNISIEILGPVNSYMIYMLKVYKLILRCFG  60
ECO0433        MKVAFLSAYDPLSTSSWSGTPYYMLKALSKRNISIEILGPVNSYMIYMLKVYKLILRCFG  60
ECO0224        MKVAFLSAYDPLSTSSWSGTPYYMLKALSKRNISIEILGPVNSYMIYMLKVYKLILRCFG  60
ECO0411        MKVAFLSAYDPLSTSSWSGTPYYMLKALSKRNISIEILGPVNSYMIYMLKVYKLILRCFG  60
ECO0431        MKVAFLSAYDPLSTSSWSGTPYYMLKALSKRNISIEILGPVNSYMIYMLKVYKLILRCFG  60
ECO0353        MKVAFLSAYDPLSTSSWSGTPYYMLKALSKRNISIEILGPVNSYMIYMLKVYKLILRCFG  60
               ************************************************************

NCTC13441      KEYDYSHSKLLSRYYGRIFGRKLKKIDGLDFIIAPAGSSQIAFLKTTIPIIYLSDTTYDQ  120
VREC1403       KEYDYSHSKLLSRYYGRIFGRKLKKIDGLDFIIAPAGSSQIAFLKTTIPIIYLSDTTYDQ  120
ECO0218        KEYDYSHSKLLSRYYGRIFGRKLKKIDGLDFIIAPAGSSQIAFLKTTIPIIYLSDTTYDQ  120
ECO0061        KEYDYSHSKLLSRYYGRIFGRKLKKIDGLDFIIAPAGSSQIAFLKTTIPIIYLSDTTYDQ  120
ECO0211        KEYDYSHSKLLSRYYGRIFGRKLKKIDGLDFIIAPAGSSQIAFLKTTIPIIYLSDTTYDQ  120
ECO0387        KEYDYSHSKLLSRYYGRIFGRKLKKIDGLDFIIAPAGSSQIAFLKTTIPIIYLSDTTYDQ  120
VRES1610       KEYDYSHSKLLSRYYGRIFGRKLKKIDGLDFIIAPAGSSQIAFLKTTIPIIYLSDTTYDQ  120
VRES1619       KEYDYSHSKLLSRYYGRIFGRKLKKIDGLDFIIAPAGSSQIAFLKTTIPIIYLSDTTYDQ  120
VRES0739       KEYDYSHSKLLSRYYGRIFGRKLKKIDGLDFIIAPAGSSQIAFLKTTIPIIYLSDTTYDQ  120
VREC0645       KEYDYSHSKLLSRYYGRIFGRKLKKIDGLDFIIAPAGSSQIAFLKTTIPIIYLSDTTYDQ  120
VRES1160       KEYDYSHSKLLSRYYGRIFGRKLKKIDGLDFIIAPAGSSQIAFLKTTIPIIYLSDTTYDQ  120
VRES0700       KEYDYSHSKLLSRYYGRIFGRKLKKIDGLDFIIAPAGSSQIAFLKTTIPIIYLSDTTYDQ  120
VREC1630       KEYDYSHSKLLSRYYGRIFGRKLKKIDGLDFIIAPAGSSQIAFLKTTIPIIYLSDTTYDQ  120
VREC1428       KEYDYSHSKLLSRYYGRIFGRKLKKIDGLDFIIAPAGSSQIAFLKTTIPIIYLSDTTYDQ  120
VREC1106       KEYDYSHSKLLSRYYGRIFGRKLKKIDGLDFIIAPAGSSQIAFLKTTIPIIYLSDTTYDQ  120
VREC1101       KEYDYSHSKLLSRYYGRIFGRKLKKIDGLDFIIAPAGSSQIAFLKTTIPIIYLSDTTYDQ  120
VREC1073       KEYDYSHSKLLSRYYGRIFGRKLKKIDGLDFIIAPAGSSQIAFLKTTIPIIYLSDTTYDQ  120
VREC1013       KEYDYSHSKLLSRYYGRIFGRKLKKIDGLDFIIAPAGSSQIAFLKTTIPIIYLSDTTYDQ  120
VREC0926       KEYDYSHSKLLSRYYGRIFGRKLKKIDGLDFIIAPAGSSQIAFLKTTIPIIYLSDTTYDQ  120
VREC0865       KEYDYSHSKLLSRYYGRIFGRKLKKIDGLDFIIAPAGSSQIAFLKTTIPIIYLSDTTYDQ  120
VREC0829       KEYDYSHSKLLSRYYGRIFGRKLKKIDGLDFIIAPAGSSQIAFLKTTIPIIYLSDTTYDQ  120
VREC0693       KEYDYSHSKLLSRYYGRIFGRKLKKIDGLDFIIAPAGSSQIAFLKTTIPIIYLSDTTYDQ  120
ECO0433        KEYDYSHSKLLSRYYGRIFGRKLKKIDGLDFIIAPAGSSQIAFLKTTIPIIYLSDTTYDQ  120
ECO0224        KEYDYSHSKLLSRYYGRIFGRKLKKIDGLDFIIAPAGSSQIAFLKTTIPIIYLSDTTYDQ  120
ECO0411        KEYDYSHSKLLSRYYGRIFGRKLKKIDGLDFIIAPAGSSQIAFLKTTIPIIYLSDTTYDQ  120
ECO0431        KEYDYSHSKLLSRYYGRIFGRKLKKIDGLDFIIAPAGSSQIAFLKTTIPIIYLSDTTYDQ  120
ECO0353        KEYDYSHSKLLSRYYGRIFGRKLKKIDGLDFIIAPAGSSQIAFLKTTIPIIYLSDTTYDQ  120
               ************************************************************

NCTC13441      LKSYYPNLNKKTIINDEDASLIERKAIEKATVVSFPSKWAMDFCRNYYRLDFDKLVEIPW  180
VREC1403       LKSYYPNLNKKTIINDEDASLIERKAIEKATVVSFPSKWAMDFCRNYYRLDFDKLVEIPW  180
ECO0218        LKSYYPNLNKKTIINDEDASLIERKAIEKATVVSFPSKWAMDFCRNYYRLDFDKLVEIPW  180
ECO0061        LKSYYPNLNKKTIINDEDASLIERKAIEKATVVSFPSKWAMDFCRNYYRLDFDKLVEIPW  180
ECO0211        LKSYYPNLNKKTIINDEDASLIERKAIEKATVVSFPSKWAMDFCRNYYRLDFDKLVEIPW  180
ECO0387        LKSYYPNLNKKTIINDEDASLIERKAIEKATVVSFPSKWAMDFCRNYYRLDFDKLVEIPW  180
VRES1610       LKSYYPNLNKKTIINDEDASLIERKAIEKATVVSFPSKWAMDFCRNYYRLDFDKLVEIPW  180
VRES1619       LKSYYPNLNKKTIINDEDASLIERKAIEKATVVSFPSKWAMDFCRNYYRLDFDKLVEIPW  180
VRES0739       LKSYYPNLNKKTIINDEDASLIERKAIEKATVVSFPSKWAMDFCRNYYRLDFDKLVEIPW  180
VREC0645       LKSYYPNLNKKTIINDEDASLIERKAIEKATVVSFPSKWAMDFCRNYYRLDFDKLVEIPW  180
VRES1160       LKSYYPNLNKKTIINDEDASLIERKAIEKATVVSFPSKWAMDFCRNYYRLDFDKLVEIPW  180
VRES0700       LKSYYPNLNKKTIINDEDASLIERKAIEKATVVSFPSKWAMDFCRNYYRLDFDKLVEIPW  180
VREC1630       LKSYYPNLNKKTIINDEDASLIERKAIEKATVVSFPSKWAMDFCRNYYRLDFDKLVEIPW  180
VREC1428       LKSYYPNLNKKTIINDEDASLIERKAIEKATVVSFPSKWAMDFCRNYYRLDFDKLVEIPW  180
VREC1106       LKSYYPNLNKKTIINDEDASLIERKAIEKATVVSFPSKWAMDFCRNYYRLDFDKLVEIPW  180
```

```
VREC1101       LKSYYPNLNKKTIINDEDASLIERKAIEKATVVSFPSKWAMDFCRNYYRLDFDKLVEIPW  180
VREC1073       LKSYYPNLNKKTIINDEDASLIERKAIEKATVVSFPSKWAMDFCRNYYRLDFDKLVEIPW  180
VREC1013       LKSYYPNLNKKTIINDEDASLIERKAIEKATVVSFPSKWAMDFCRNYYRLDFDKLVEIPW  180
VREC0926       LKSYYPNLNKKTIINDEDASLIERKAIEKATVVSFPSKWAMDFCRNYYRLDFDKLVEIPW  180
VREC0865       LKSYYPNLNKKTIINDEDASLIERKAIEKATVVSFPSKWAMDFCRNYYRLDFDKLVEIPW  180
VREC0829       LKSYYPNLNKKTIINDEDASLIERKAIEKATVVSFPSKWAMDFCRNYYRLDFDKLVEIPW  180
VREC0693       LKSYYPNLNKKTIINDEDASLIERKAIEKATVVSFPSKWAMDFCRNYYRLDFDKLVEIPW  180
ECO0433        LKSYYPNLNKKTIINDEDASLIERKAIEKATVVSFPSKWAMDFCRNYYRLDFDKLVEIPW  180
ECO0224        LKSYYPNLNKKTIINDEDASLIERKAIEKATVVSFPSKWAMDFCRNYYRLDFDKLVEIPW  180
ECO0411        LKSYYPNLNKKTIINDEDASLIERKAIEKATVVSFPSKWAMDFCRNYYRLDFDKLVEIPW  180
ECO0431        LKSYYPNLNKKTIINDEDASLIERKAIEKATVVSFPSKWAMDFCRNYYRLDFDKLVEIPW  180
ECO0353        LKSYYPNLNKKTIINDEDASLIERKAIEKATVVSFPSKWAMDFCRNYYRLDFDKLVEIPW  180
               ************************************************************

NCTC13441      GANLFDDIHFANKNIIQKNSYTCLFLGVDWERKGGKTALKAIEYVRQLYGIDVRLKICGC  240
VREC1403       GANLFDDIHFANKNIIQKNSYTCLFLGVDWERKGGKTALKAIEYVRQLYGIDVRLKICGC  240
ECO0218        GANLFDDIHFANKNIIQKNSYTCLFLGVDWERKGGKTALKAIEYVRQLYGIDVRLKICGC  240
ECO0061        GANLFDDIHFANKNIIQKNSYTCLFLGVDWERKGGKTALKAIEYVRQLYGIDVRLKICGC  240
ECO0211        GANLFDDIHFANKNIIQKNSYTCLFLGVDWERKGGKTALKAIEYVRQLYGIDVRLKICGC  240
ECO0387        GANLFDDIHFANKNIIQKNSYTCLFLGVDWERKGGKTALKAIEYVRQLYGIDVRLKICGC  240
VRES1610       GANLFDDIHFANKNIIQKNSYTCLFLGVDWERKGGKTALKAIEYVRQLYGIDVRLKICGC  240
VRES1619       GANLFDDIHFANKNIIQKNSYTCLFLGVDWERKGGKTALKAIEYVRQLYGIDVRLKICGC  240
VRES0739       GANLFDDIHFANKNIIQKNSYTCLFLGVDWERKGGKTALKAIEYVRQLYGIDVRLKICGC  240
VREC0645       GANLFDDIHFANKNIIQKNSYTCLFLGVDWERKGGKTALKAIEYVRQLYGIDVRLKICGC  240
VRES1160       GANLFDDIHFANKNIIQKNSYTCLFLGVDWERKGGKTALKAIEYVRQLYGIDVRLKICGC  240
VRES0700       GANLFDDIHFANKNIIQKNSYTCLFLGVDWERKGGKTALKAIEYVRQLYGIDVRLKICGC  240
VREC1630       GANLFDDIHFANKNIIQKNSYTCLFLGVDWERKGGKTALKAIEYVRQLYGIDVRLKICGC  240
VREC1428       GANLFDDIHFANKNIIQKNSYTCLFLGVDWERKGGKTALKAIEYVRQLYGIDVRLKICGC  240
VREC1106       GANLFDDIHFANKNIIQKNSYTCLFLGVDWERKGGKTALKAIEYVRQLYGIDVRLKICGC  240
VREC1101       GANLFDDIHFANKNIIQKNSYTCLFLGVDWERKGGKTALKAIEYVRQLYGIDVRLKICGC  240
VREC1073       GANLFDDIHFANKNIIQKNSYTCLFLGVDWERKGGKTALKAIEYVRQLYGIDVRLKICGC  240
VREC1013       GANLFDDIHFANKNIIQKNSYTCLFLGVDWERKGGKTALKAIEYVRQLYGIDVRLKICGC  240
VREC0926       GANLFDDIHFANKNIIQKNSYTCLFLGVDWERKGGKTALKAIEYVRQLYGIDVRLKICGC  240
VREC0865       GANLFDDIHFANKNIIQKNSYTCLFLGVDWERKGGKTALKAIEYVRQLYGIDVRLKICGC  240
VREC0829       GANLFDDIHFANKNIIQKNSYTCLFLGVDWERKGGKTALKAIEYVRQLYGIDVRLKICGC  240
VREC0693       GANLFDDIHFANKNIIQKNSYTCLFLGVDWERKGGKTALKAIEYVRQLYGIDVRLKICGC  240
ECO0433        GANLFDDIHFANKNIIQKNSYTCLFLGVDWERKGGKTALKAIEYVRQLYGIDVRLKICGC  240
ECO0224        GANLFDDIHFANKNIIQKNSYTCLFLGVDWERKGGKTALKAIEYVRQLYGIDVRLKICGC  240
ECO0411        GANLFDDIHFANKNIIQKNSYTCLFLGVDWERKGGKTALKAIEYVRQLYGIDVRLKICGC  240
ECO0431        GANLFDDIHFANKNIIQKNSYTCLFLGVDWERKGGKTALKAIEYVRQLYGIDVRLKICGC  240
ECO0353        GANLFDDIHFANKNIIQKNSYTCLFLGVDWERKGGKTALKAIEYVRQLYGIDVRLKICGC  240
               ************************************************************

NCTC13441      TPNQKILPTWVELIDKVDKNNVDEYQKFIDVLSNADILLLPTIAECYGMVFCEAAAFGLP  300
VREC1403       TPNQKILPTWVELIDKVDKNNVDEYQKFIDVLSNADILLLPTIAECYGMVFCEAAAFGLP  300
ECO0218        TPNQKILPTWVELIDKVDKNNVDEYQKFIDVLSNADILLLPTIAECYGMVFCEAAAFGLP  300
ECO0061        TPNQKILPTWVELIDKVDKNNVDEYQKFIDVLSNADILLLPTIAECYGMVFCEAAAFGLP  300
ECO0211        TPNQKILPTWVELIDKVDKNNVDEYQKFIDVLSNADILLLPTIAECYGMVFCEAAAFGLP  300
ECO0387        TPNQKILPTWVELIDKVDKNNVDEYQKFIDVLSNADILLLPTIAECYGMVFCEAAAFGLP  300
VRES1610       TPNQKILPTWVELIDKVDKNNVDEYQKFIDVLSNADILLLPTIAECYGMVFCEAAAFGLP  300
VRES1619       TPNQKILPTWVELIDKVDKNNVDEYQKFIDVLSNADILLLPTIAECYGMVFCEAAAFGLP  300
VRES0739       TPNQKILPTWVELIDKVDKNNVDEYQKFIDVLSNADILLLPTIAECYGMVFCEAAAFGLP  300
VREC0645       TPNQKILPTWVELIDKVDKNNVDEYQKFIDVLSNADILLLPTIAECYGMVFCEAAAFGLP  300
VRES1160       TPNQKILPTWVELIDKVDKNNVDEYQKFIDVLSNADILLLPTIAECYGMVFCEAAAFGLP  300
VRES0700       TPNQKILPTWVELIDKVDKNNVDEYQKFIDVLSNADILLLPTIAECYGMVFCEAAAFGLP  300
VREC1630       TPNQKILPTWVELIDKVDKNNVDEYQKFIDVLSNADILLLPTIAECYGMVFCEAAAFGLP  300
VREC1428       TPNQKILPTWVELIDKVDKNNVDEYQKFIDVLSNADILLLPTIAECYGMVFCEAAAFGLP  300
VREC1106       TPNQKILPTWVELIDKVDKNNVDEYQKFIDVLSNADILLLPTIAECYGMVFCEAAAFGLP  300
VREC1101       TPNQKILPTWVELIDKVDKNNVDEYQKFIDVLSNADILLLPTIAECYGMVFCEAAAFGLP  300
VREC1073       TPNQKILPTWVELIDKVDKNNVDEYQKFIDVLSNADILLLPTIAECYGMVFCEAAAFGLP  300
VREC1013       TPNQKILPTWVELIDKVDKNNVDEYQKFIDVLSNADILLLPTIAECYGMVFCEAAAFGLP  300
VREC0926       TPNQKILPTWVELIDKVDKNNVDEYQKFIDVLSNADILLLPTIAECYGMVFCEAAAFGLP  300
VREC0865       TPNQKILPTWVELIDKVDKNNVDEYQKFIDVLSNADILLLPTIAECYGMVFCEAAAFGLP  300
VREC0829       TPNQKILPTWVELIDKVDKNNVDEYQKFIDVLSNADILLLPTIAECYGMVFCEAAAFGLP  300
VREC0693       TPNQKILPTWVELIDKVDKNNVDEYQKFIDVLSNADILLLPTIAECYGMVFCEAAAFGLP  300
ECO0433        TPNQKILPTWVELIDKVDKNNVDEYQKFIDVLSNADILLLPTIAECYGMVFCEAAAFGLP  300
ECO0224        TPNQKILPTWVELIDKVDKNNVDEYQKFIDVLSNADILLLPTIAECYGMVFCEAAAFGLP  300
ECO0411        TPNQKILPTWVELIDKVDKNNVDEYQKFIDVLSNADILLLPTIAECYGMVFCEAAAFGLP  300
ECO0431        TPNQKILPTWVELIDKVDKNNVDEYQKFIDVLSNADILLLPTIAECYGMVFCEAAAFGLP  300
ECO0353        TPNQKILPTWVELIDKVDKNNVDEYQKFIDVLSNADILLLPTIAECYGMVFCEAAAFGLP  300
               ************************************************************

NCTC13441      VVATDTGGVSSIVINERTGILIKDPLDYKHFGNAIHKIISSVETYQNYSQNARIRYNNIL  360
VREC1403       VVATDTGGVSSIVINERTGILIKDPLDYKHFGNAIHKIISSVETYQNYSQNARIRYNNIL  360
ECO0218        VVATDTGGVSSIVINERTGILIKDPLDYKHFGNAIHKIISSVETYQNYSQNARIRYNNIL  360
ECO0061        VVATDTGGVSSIVINERTGILIKDPLDYKHFGNAIHKIISSVETYQNYSQNARIRYNNIL  360
ECO0211        VVATDTGGVSSIVINERTGILIKDPLDYKHFGNAIHKIISSVETYQNYSQNARIRYNNIL  360
```

```
ECO0387         VVATDTGGVSSIVINERTGILIKDPLDYKHFGNAIHKIISSVETYQNYSQNARIRYNNIL  360
VRES1610        VVATDTGGVSSIVINERTGILIKDPLDYKHFGNAIHKIISSVETYQNYSQNARIRYNNIL  360
VRES1619        VVATDTGGVSSIVINERTGILIKDPLDYKHFGNAIHKIISSVETYQNYSQNARIRYNNIL  360
VRES0739        VVATDTGGVSSIVINERTGILIKDPLDYKHFGNAIHKIISSVETYQNYSQNARIRYNNIL  360
VREC0645        VVATDTGGVSSIVINERTGILIKDPLDYKHFGNAIHKIISSVETYQN-------------  347
VRES1160        VVATDTGGVSSIVINERTGILIKDPLDYKHFGNAIHKIISSVETYQNYSQNARIRYNNIL  360
VRES0700        VVATDTGGVSSIVINERTGILIKDPLDYKHFGNAIHKIISSVETYQNYSQNARIRYNNIL  360
VREC1630        VVATDTGGVSSIVINERTGILIKDPLDYKHFGNAIHKIISSVETYQNYSQNARIRYNNIL  360
VREC1428        VVATDTGGVSSIVINERTGILIKDPLDYKHFGNAIHKIISSVETYQNYSQNARIRYNNIL  360
VREC1106        VVATDTGGVSSIVINERTGILIKDPLDYKHFGNAIHKIISSVETYQNYSQNARIRYNNIL  360
VREC1101        VVATDTGGVSSIVINERTGILIKDPLDYKHFGNAIHKIISSVETYQNYSQNARIRYNNIL  360
VREC1073        VVATDTGGVSSIVINERTGILIKDPLDYKHFGNAIHKIISSVETYQNYSQNARIRYNNIL  360
VREC1013        VVATDTGGVSSIVINERTGILIKDPLDYKHFGNAIHKIISSVETYQNYSQNARIRYNNIL  360
VREC0926        VVATDTGGVSSIVINERTGILIKDPLDYKHFGNAIHKIISSVETYQNYSQNARIRYNNIL  360
VREC0865        VVATDTGGVSSIVINERTGILIKDPLDYKHFGNAIHKIISSVETYQNYSQNARIRYNNIL  360
VREC0829        VVATDTGGVSSIVINERTGILIKDPLDYKHFGNAIHKIISSVETYQNYSQNARIRYNNIL  360
VREC0693        VVATDTGGVSSIVINERTGILIKDPLDYKHFGNAIHKIISSVETYQNYSQNARIRYNNIL  360
ECO0433         VVATDTGGVSSIVINERTGILIKDPLDYKHFGNAIHKIISSVETYQNYSQNARIRYNNIL  360
ECO0224         VVATDTGGVSSIVINERTGILIKDPLDYKHFGNAIHKIISSVETYQNYSQNARIRYNNIL  360
ECO0411         VVATDTGGVSSIVINERTGILIKDPLDYKHFGNAIHKIISSVETYQNYSQNARIRYNNIL  360
ECO0431         VVATDTGGVSSIVINERTGILIKDPLDYKHFGNAIHKIISSVETYQNYSQNARIRYNNIL  360
ECO0353         VVATDTGGVSSIVINERTGILIKDPLDYKHFGNAIHKIISSVETYQNYSQNARIRYNNIL  360
                ***********************************************

NCTC13441       HWDNWAKKIIEIMYEHKNRRIK  382
VREC1403        HWDNWAKKIIEIMYEHKNRRIK  382
ECO0218         HWDNWAKKIIEIMYEHKNRRIK  382
ECO0061         HWDNWAKKIIEIMYEHKNRRIK  382
ECO0211         HWDNWAKKIIEIMYEHKNRRIK  382
ECO0387         HWDNWAKKIIEIMYEHKNRRIK  382
VRES1610        HWDNWAKKIIEIMYEHKNRRIK  382
VRES1619        HWDNWAKKIIEIMYEHKNRRIK  382
VRES0739        HWDNWAKKIIEIMYEHKNRRIK  382
VREC0645        ----------------------  347
VRES1160        HWDNWAKKIIEIMYEHKNRRIK  382
VRES0700        HWDNWAKKIIEIMYEHKNRRIK  382
VREC1630        HWDNWAKKIIEIMYEHKNRRIK  382
VREC1428        HWDNWAKKIIEIMYEHKNRRIK  382
VREC1106        HWDNWAKKIIEIMYEHKNRRIK  382
VREC1101        HWDNWAKKIIEIMYEHKNRRIK  382
VREC1073        HWDNWAKKIIEIMYEHKNRRIK  382
VREC1013        HWDNWAKKIIEIMYEHKNRRIK  382
VREC0926        HWDNWAKKIIEIMYEHKNRRIK  382
VREC0865        HWDNWAKKIIEIMYEHKNRRIK  382
VREC0829        HWDNWAKKIIEIMYEHKNRRIK  382
VREC0693        HWDNWAKKIIEIMYEHKNRRIK  382
ECO0433         HWDNWAKKIIEIMYEHKNRRIK  382
ECO0224         HWDNWAKKIIEIMYEHKNRRIK  382
ECO0411         HWDNWAKKIIEIMYEHKNRRIK  382
ECO0431         HWDNWAKKIIEIMYEHKNRRIK  382
ECO0353         HWDNWAKKIIEIMYEHKNRRIK  382
```

CLUSTAL O(1.2.4) multiple sequence alignment - **Wzy**

```
ECO0353          MSIRIEESNSTKRIICLFILFLVFPDFLFYTLGVDNFSISTIISITLLFVFLRAKNICKD        60
ECO0431          MSIRIEESNSTKRIICLFILFLVFPDFLFYTLGVDNFSISTIISITLLFVFLRAKNICKD        60
ECO0411          MSIRIEESNSTKRIICLFILFLVFPDFLFYTLGVDNFSISTIISITLLFVFLRAKNICKD        60
ECO0224          MSIRIEESNSTKRIICLFILFLVFPDFLFYTLGVDNFSISTIISITLLFVFLRAKNICKD        60
ECO0433          MSIRIEESNSTKRIICLFILFLVFPDFLFYTLGVDNFSISTIISITLLFVFLRAKNICKD        60
VREC0693         MSIRIEESNSTKRIICLFILFLVFPDFLFYTLGVDNFSISTIISITLLFVFLRAKNICKD        60
VREC0829         MSIRIEESNSTKRIICLFILFLVFPDFLFYTLGVDNFSISTIISITLLFVFLRAKNICKD        60
VREC0865         MSIRIEESNSTKRIICLFILFLVFPDFLFYTLGVDNFSISTIISITLLFVFLRAKNICKD        60
VREC0926         MSIRIEESNSTKRIICLFILFLVFPDFLFYTLGVDNFSISTIISITLLFVFLRAKNICKD        60
VREC1013         MSIRIEESNSTKRIICLFILFLVFPDFLFYTLGVDNFSISTIISITLLFVFLRAKNICKD        60
VREC1073         MSIRIEESNSTKRIICLFILFLVFPDFLFYTLGVDNFSISTIISITLLFVFLRAKNICKD        60
VREC1101         MSIRIEESNSTKRIICLFILFLVFPDFLFYTLGVDNFSISTIISITLLFVFLRAKNICKD        60
VREC1106         MSIRIEESNSTKRIICLFILFLVFPDFLFYTLGVDNFSISTIISITLLFVFLRAKNICKD        60
VREC1428         MSIRIEESNSTKRIICLFILFLVFPDFLFYTLGVDNFSISTIISITLLFVFLRAKNICKD        60
VREC1630         MSIRIEESNSTKRIICLFILFLVFPDFLFYTLGVDNFSISTIISITLLFVFLRAKNICKD        60
```

```
VRES0700        MSIRIEESNSTKRIICLFILFLVFPDFLFYTLGVDNFSISTIISITLLFVFLRAKNICKD        60
VRES1160        MSIRIEESNSTKRIICLFILFLVFPDFLFYTLGVDNFSISTIISITLLFVFLRAKNICKD        60
VREC0645        MSIRIEESNSTKRIICLFILFLVFPDFLFYTLGVDNFSISTIISITLLFVFLRAKNICKD        60
VRES0739        MSIRIEESNSTKRIICLFILFLVFPDFLFYTLGVDNFSISTIISITLLFVFLRAKNICKD        60
VRES1619        MSIRIEESNSTKRIICLFILFLVFPDFLFYTLGVDNFSISTIISITLLFVFLRAKNICKD        60
VRES1610        MSIRIEESNSTKRIICLFILFLVFPDFLFYTLGVDNFSISTIISITLLFVFLRAKNICKD        60
ECO0211         MSIRIEESNSTKRIICLFILFLVFPDFLFYTLGVDNFSISTIISITLLFVFLRAKNICKD        60
ECO0061         MSIRIEESNSTKRIICLFILFLVFPDFLFYTLGVDNFSISTIISITLLFVFLRAKNICKD        60
VREC1403        MSIRIEESNSTKRIICLFILFLVFPDFLFYTLGVDNFSISTIISITLLFVFLRAKNICKD        60
NCTC13441       MSIRIEESNSTKRIICLFILFLVFPDFLFYTLGVDNFSISTIISITLLFVFLRAKNICKD        60
ECO0218orf2     ------------------------------------------------------------        0
ECO0387orf2     ------------------------------------------------------------        0
ECO0218orf1     MSIRIEESNSTKRIICLFILFLVFPDFLFYTLGVDNFSISTIISITLLFVFLRAKNICKD        60
ECO0387orf1     MSIRIEESNSTKRIICLFILFLVFPDFLFYTLGVDNFSISTIISITLLFVFLRAKNICKD        60

ECO0353         NFLIIVALFILLCFNCLLSMLFNIEQALTFKVVLSIYSILIMAYVSSCYAQTLWLCSEEI        120
ECO0431         NFLIIVALFILLCFNCLLSMLFNIEQALTFKVVLSIYSILIMAYVSSCYAQTLWLCSEEI        120
ECO0411         NFLIIVALFILLCFNCLLSMLFNIEQALTFKVVLSIYSILIMAYVSSCYAQTLWLCSEEI        120
ECO0224         NFLIIVALFILLCFNCLLSMLFNIEQALTFKVVLSIYSILIMAYVSSCYAQTLWLCSEEI        120
ECO0433         NFLIIVALFILLCFNCLLSMLFNIEQALTFKVVLSIYSILIMAYVSSCYAQTLWLCSEEI        120
VREC0693        NFLIIVALFILLCFNCLLSMLFNIEQALTFKVVLSIYSILIMAYVSSCYAQTLWLCSEEI        120
VREC0829        NFLIIVALFILLCFNCLLSMLFNIEQALTFKVVLSIYSILIMAYVSSCYAQTLWLCSEEI        120
VREC0865        NFLIIVALFILLCFNCLLSMLFNIEQALTFKVVLSIYSILIMAYVSSCYAQTLWLCSEEI        120
VREC0926        NFLIIVALFILLCFNCLLSMLFNIEQALTFKVVLSIYSILIMAYVSSCYAQTLWLCSEEI        120
VREC1013        NFLIIVALFILLCFNCLLSMLFNIEQALTFKVVLSIYSILIMAYVSSCYAQTLWLCSEEI        120
VREC1073        NFLIIVALFILLCFNCLLSMLFNIEQALTFKVVLSIYSILIMAYVSSCYAQTLWLCSEEI        120
VREC1101        NFLIIVALFILLCFNCLLSMLFNIEQALTFKVVLSIYSILIMAYVSSCYAQTLWLCSEEI        120
VREC1106        NFLIIVALFILLCFNCLLSMLFNIEQALTFKVVLSIYSILIMAYVSSCYAQTLWLCSEEI        120
VREC1428        NFLIIVALFILLCFNCLLSMLFNIEQALTFKVVLSIYSILIMAYVSSCYAQTLWLCSEEI        120
VREC1630        NFLIIVALFILLCFNCLLSMLFNIEQALTFKVVLSIYSILIMAYVSSCYAQTLWLCSEEI        120
VRES0700        NFLIIVALFILLCFNCLLSMLFNIEQALTFKVVLSIYSILIMAYVSSCYAQTLWLCSEEI        120
VRES1160        NFLIIVALFILLCFNCLLSMLFNIEQALTFKVVLSIYSILIMAYVSSCYAQTLWLCSEEI        120
VREC0645        NFLIIVALFILLCFNCLLSMLFNIEQALTFKVVLSIYSILIMAYVSSCYAQTLWLCSEEI        120
VRES0739        NFLIIVALFILLCFNCLLSMLFNIEQALTFKVVLSIYSILIMAYVSSCYAQTLWLCSEEI        120
VRES1619        NFLIIVALFILLCFNCLLSMLFNIEQALTFKVVLSIYSILIMAYVSSCYAQTLWLCSEEI        120
VRES1610        NFLIIVALFILLCFNCLLSMLFNIEQALTFKVVLSIYSILIMAYVSSCYAQTLWLCSEEI        120
ECO0211         NFLIIVALFILLCFNCLLSMLFNIEQALTFKVVLSIYSILIMAYVSSCYAQTLWLCSEEI        120
ECO0061         NFLIIVALFILLCFNCLLSMLFNIEQALTFKVVLSIYSILIMAYVSSCYAQTLWLCSEEI        120
VREC1403        NFLIIVALFILLCFNCLLSMLFNIEQALTFKVVLSIYSILIMAYVSSCYAQTLWLCSEEI        120
NCTC13441       NFLIIVALFILLCFNCLLSMLFNIEQALTFKVVLSIYSILIMAYVSSCYAQTLWLCSEEI        120
ECO0218orf2     ------------------------------------------------------------        0
ECO0387orf2     ------------------------------------------------------------        0
ECO0218orf1     NFLIIVALFILLCFNCLLSMLFNIEQALTFKVVLSIYSILIMAYVSSCYAQTLWLCSEEI        120
ECO0387orf1     NFLIIVALFILLCFNCLLSMLFNIEQALTFKVVLSIYSILIMAYVSSCYAQTLWLCSEEI        120

ECO0353         LKRSVFYLFAFLCLIGIISILLQKTEIIHDKSMILFPEPSAFALVFIPIFSFCLYYTRGG        180
ECO0431         LKRSVFYLFAFLCLIGIISILLQKTEIIHDKSMILFPEPSAFALVFIPIFSFCLYYTRGG        180
ECO0411         LKRSVFYLFAFLCLIGIISILLQKTEIIHDKSMILFPEPSAFALVFIPIFSFCLYYTRGG        180
ECO0224         LKRSVFYLFAFLCLIGIISILLQKTEIIHDKSMILFPEPSAFALVFIPIFSFCLYYTRGG        180
ECO0433         LKRSVFYLFAFLCLIGIISILLQKTEIIHDKSMILFPEPSAFALVFIPIFSFCLYYTRGG        180
VREC0693        LKRSVFYLFAFLCLIGIISILLQKTEIIHDKSMILFPEPSAFALVFIPIFSFCLYYTRGG        180
VREC0829        LKRSVFYLFAFLCLIGIISILLQKTEIIHDKSMILFPEPSAFALVFIPIFSFCLYYTRGG        180
VREC0865        LKRSVFYLFAFLCLIGIISILLQKTEIIHDKSMILFPEPSAFALVFIPIFSFCLYYTRGG        180
VREC0926        LKRSVFYLFAFLCLIGIISILLQKTEIIHDKSMILFPEPSAFALVFIPIFSFCLYYTRGG        180
VREC1013        LKRSVFYLFAFLCLIGIISILLQKTEIIHDKSMILFPEPSAFALVFIPIFSFCLYYTRGG        180
VREC1073        LKRSVFYLFAFLCLIGIISILLQKTEIIHDKSMILFPEPSAFALVFIPIFSFCLYYTRGG        180
VREC1101        LKRSVFYLFAFLCLIGIISILLQKTEIIHDKSMILFPEPSAFALVFIPIFSFCLYYTRGG        180
VREC1106        LKRSVFYLFAFLCLIGIISILLQKTEIIHDKSMILFPEPSAFALVFIPIFSFCLYYTRGG        180
VREC1428        LKRSVFYLFAFLCLIGIISILLQKTEIIHDKSMILFPEPSAFALVFIPIFSFCLYYTRGG        180
VREC1630        LKRSVFYLFAFLCLIGIISILLQKTEIIHDKSMILFPEPSAFALVFIPIFSFCLYYTRGG        180
VRES0700        LKRSVFYLFAFLCLIGIISILLQKTEIIHDKSMILFPEPSAFALVFIPIFSFCLYYTRGG        180
VRES1160        LKRSVFYLFAFLCLIGIISILLQKTEIIHDKSMILFPEPSAFALVFIPIFSFCLYYTRGG        180
VREC0645        LKRSVFYLFAFLCLIGIISILLQKTEIIHDKSMILFPEPSAFALVFIPIFSFCLYYTRGG        180
VRES0739        LKRSVFYLFAFLCLIGIISILLQKTEIIHDKSMILFPEPSAFALVFIPIFSFCLYYTRGG        180
VRES1619        LKRSVFYLFAFLCLIGIISILLQKTEIIHDKSMILFPEPSAFALVFIPIFSFCLYYTRGG        180
VRES1610        LKRSVFYLFAFLCLIGIISILLQKTEIIHDKSMILFPEPSAFALVFIPIFSFCLYYTRGG        180
ECO0211         LKRSVFYLFAFLCLIGIISILLQKTEIIHDKSMILFPEPSAFALVFIPIFSFCLYYTRGG        180
ECO0061         LKRSVFYLFAFLCLIGIISILLQKTEIIHDKSMILFPEPSAFALVFIPIFSFCLYYTRGG        180
VREC1403        LKRSVFYLFAFLCLIGIISILLQKTEIIHDKSMILFPEPSAFALVFIPIFSFCLYYTRGG        180
NCTC13441       LKRSVFYLFAFLCLIGIISILLQKTEIIHDKSMILFPEPSAFALVFIPIFSFCLYYTRGG        180
ECO0218orf2     ------------------------------------------------------------        0
ECO0387orf2     ------------------------------------------------------------        0
ECO0218orf1     LKRSVFYLFAFLCLIGIISILLQKTEIIHDKSMILFPEPSAFALVFIPIFSFCLYYTRGG        180
ECO0387orf1     LKRSVFYLFAFLCLIGIISILLQKTEIIHDKSMILFPEPSAFALVFIPIFSFCLYYTRGG        180
```

| | | |
|---|---|---|
| ECO0353 | GLLLLYILSLGIALGIQNLTMLVGIVISVFVMKKITIRQTIVILLGAWIFSMILSDLDIS | 240 |
| ECO0431 | GLLLLYILSLGIALGIQNLTMLVGIVISVFVMKKITIRQTIVILLGAWIFSMILSDLDIS | 240 |
| ECO0411 | GLLLLYILSLGIALGIQNLTMLVGIVISVFVMKKITIRQTIVILLGAWIFSMILSDLDIS | 240 |
| ECO0224 | GLLLLYILSLGIALGIQNLTMLVGIVISVFVMKKITIRQTIVILLGAWIFSMILSDLDIS | 240 |
| ECO0433 | GLLLLYILSLGIALGIQNLTMLVGIVISVFVMKKITIRQTIVILLGAWIFSMILSDLDIS | 240 |
| VREC0693 | GLLLLYILSLGIALGIQNLTMLVGIVISVFVMKKITIRQTIVILLGAWIFSMILSDLDIS | 240 |
| VREC0829 | GLLLLYILSLGIALGIQNLTMLVGIVISVFVMKKITIRQTIVILLGAWIFSMILSDLDIS | 240 |
| VREC0865 | GLLLLYILSLGIALGIQNLTMLVGIVISVFVMKKITIRQTIVILLGAWIFSMILSDLDIS | 240 |
| VREC0926 | GLLLLYILSLGIALGIQNLTMLVGIVISVFVMKKITIRQTIVILLGAWIFSMILSDLDIS | 240 |
| VREC1013 | GLLLLYILSLGIALGIQNLTMLVGIVISVFVMKKITIRQTIVILLGAWIFSMILSDLDIS | 240 |
| VREC1073 | GLLLLYILSLGIALGIQNLTMLVGIVISVFVMKKITIRQTIVILLGAWIFSMILSDLDIS | 240 |
| VREC1101 | GLLLLYILSLGIALGIQNLTMLVGIVISVFVMKKITIRQTIVILLGAWIFSMILSDLDIS | 240 |
| VREC1106 | GLLLLYILSLGIALGIQNLTMLVGIVISVFVMKKITIRQTIVILLGAWIFSMILSDLDIS | 240 |
| VREC1428 | GLLLLYILSLGIALGIQNLTMLVGIVISVFVMKKITIRQTIVILLGAWIFSMILSDLDIS | 240 |
| VREC1630 | GLLLLYILSLGIALGIQNLTMLVGIVISVFVMKKITIRQTIVILLGAWIFSMILSDLDIS | 240 |
| VRES0700 | GLLLLYILSLGIALGIQNLTMLVGIVISVFVMKKITIRQTIVILLGAWIFSMILSDLDIS | 240 |
| VRES1160 | GLLLLYILSLGIALGIQNLTMLVGIVISVFVMKKITIRQTIVILLGAWIFSMILSDLDIS | 240 |
| VREC0645 | GLLLLYILSLGIALGIQNLTMLVGIVISVFVMKKITIRQTIVILLGAWIFSMILSDLDIS | 240 |
| VRES0739 | GLLLLYILSLGIALGIQNLTMLVGIVISVFVMKKITIRQTIVILLGAWIFSMILSDLDIS | 240 |
| VRES1619 | GLLLLYILSLGIALGIQNLTMLVGIVISVFVMKKITIRQTIVILLGAWIFSMILSDLDIS | 240 |
| VRES1610 | GLLLLYILSLGIALGIQNLTMLVGIVISVFVMKKITIRQTIVILLGAWIFSMILSDLDIS | 240 |
| ECO0211 | GLLLLYILSLGIALGIQNLTMLVGIVISVFVMKKITIRQTIVILLGAWIFSMILSDLDIS | 240 |
| ECO0061 | GLLLLYILSLGIALGIQNLTMLVGIVISVFVMKKITIRQTIVILLGAWIFSMILSDLDIS | 240 |
| VREC1403 | GLLLLYILSLGIALGIQNLTMLVGIVISVFVMKKITIRQTIVILLGAWIFSMILSDLDIS | 240 |
| NCTC13441 | GLLLLYILSLGIALGIQNLTMLVGIVISVFVMKKITIRQTIVILLGAWIFSMILSDLDIS | 240 |
| ECO0218orf2 | ---------------------------------------------------MILSDLDIS | 9 |
| ECO0387orf2 | --------------------MLVGIVISVFVMKKITIRQTIVILLGAWIFSMILSDLDIS | 40 |
| ECO0218orf1 | GATIALYIIFGYCVRYPEFNNVGRH-CD-------------------------------- | 207 |
| ECO0387orf1 | GYYCSIYYLWVLR----------------------------------------------- | 193 |

| | | |
|---|---|---|
| ECO0353 | YYTSRLDFKNTTNLSVLVYLSGIERAFLNFITSYGLGIGFQQMGVNGEIGIYQQILAELD | 300 |
| ECO0431 | YYTSRLDFKNTTNLSVLVYLSGIERAFLNFITSYGLGIGFQQMGVNGEIGIYQQILAELD | 300 |
| ECO0411 | YYTSRLDFKNTTNLSVLVYLSGIERAFLNFITSYGLGIGFQQMGVNGEIGIYQQILAELD | 300 |
| ECO0224 | YYTSRLDFKNTTNLSVLVYLSGIERAFLNFITSYGLGIGFQQMGVNGEIGIYQQILAELD | 300 |
| ECO0433 | YYTSRLDFKNTTNLSVLVYLSGIERAFLNFITSYGLGIGFQQMGVNGEIGIYQQILAELD | 300 |
| VREC0693 | YYTSRLDFKNTTNLSVLVYLSGIERAFLNFITSYGLGIGFQQMGVNGEIGIYQQILAELD | 300 |
| VREC0829 | YYTSRLDFKNTTNLSVLVYLSGIERAFLNFITSYGLGIGFQQMGVNGEIGIYQQILAELD | 300 |
| VREC0865 | YYTSRLDFKNTTNLSVLVYLSGIERAFLNFITSYGLGIGFQQMGVNGEIGIYQQILAELD | 300 |
| VREC0926 | YYTSRLDFKNTTNLSVLVYLSGIERAFLNFITSYGLGIGFQQMGVNGEIGIYQQILAELD | 300 |
| VREC1013 | YYTSRLDFKNTTNLSVLVYLSGIERAFLNFITSYGLGIGFQQMGVNGEIGIYQQILAELD | 300 |
| VREC1073 | YYTSRLDFKNTTNLSVLVYLSGIERAFLNFITSYGLGIGFQQMGVNGEIGIYQQILAELD | 300 |
| VREC1101 | YYTSRLDFKNTTNLSVLVYLSGIERAFLNFITSYGLGIGFQQMGVNGEIGIYQQILAELD | 300 |
| VREC1106 | YYTSRLDFKNTTNLSVLVYLSGIERAFLNFITSYGLGIGFQQMGVNGEIGIYQQILAELD | 300 |
| VREC1428 | YYTSRLDFKNTTNLSVLVYLSGIERAFLNFITSYGLGIGFQQMGVNGEIGIYQQILAELD | 300 |
| VREC1630 | YYTSRLDFKNTTNLSVLVYLSGIERAFLNFITSYGLGIGFQQMGVNGEIGIYQQILAELD | 300 |
| VRES0700 | YYTSRLDFKNTTNLSVLVYLSGIERAFLNFITSYGLGIGFQQMGVNGEIGIYQQILAELD | 300 |
| VRES1160 | YYTSRLDFKNTTNLSVLVYLSGIERAFLNFITSYGLGIGFQQMGVNGEIGIYQQILAELD | 300 |
| VREC0645 | YYTSRLDFKNTTNLSVLVYLSGIERAFLNFITSYGLGIGFQQMGVNGEIGIYQQILAELD | 300 |
| VRES0739 | YYTSRLDFKNTTNLSVLVYLSGIERAFLNFITSYGLGIGFQQMGVNGEIGIYQQILAELD | 300 |
| VRES1619 | YYTSRLDFKNTTNLSVLVYLSGIERAFLNFITSYGLGIGFQQMGVNGEIGIYQQILAELD | 300 |
| VRES1610 | YYTSRLDFKNTTNLSVLVYLSGIERAFLNFITSYGLGIGFQQMGVNGEIGIYQQILAELD | 300 |
| ECO0211 | YYTSRLDFKNTTNLSVLVYLSGIERAFLNFITSYGLGIGFQQMGVNGEIGIYQQILAELD | 300 |
| ECO0061 | YYTSRLDFKNTTNLSVLVYLSGIERAFLNFITSYGLGIGFQQMGVNGEIGIYQQILAELD | 300 |
| VREC1403 | YYTSRLDFKNTTNLSVLVYLSGIERAFLNFITSYGLGIGFQQMGVNGEIGIYQQILAELD | 300 |
| NCTC13441 | YYTSRLDFKNTTNLSVLVYLSGIERAFLNFITSYGLGIGFQQMGVNGEIGIYQQILAELD | 300 |
| ECO0218orf2 | YYTSRLDFKNTTNLSVLVYLSGIERAFLNFITSYGLGIGFQQMGVNGEIGIYQQILAELD | 69 |
| ECO0387orf2 | YYTSRLDFKNTTNLSVLVYLSGIERAFLNFITSYGLGIGFQQMGVNGEIGIYQQILAELD | 100 |
| ECO0218orf1 | ------------------------------------------------------------ | 207 |
| ECO0387orf1 | ------------------------------------------------------------ | 193 |

| | | |
|---|---|---|
| ECO0353 | APMLNIYDGSFISSKLISEFGVIGALMCIFYFFYFSRFYLRFKKSKRYSPQYILAYSFYM | 360 |
| ECO0431 | APMLNIYDGSFISSKLISEFGVIGALMCIFYFFYFSRFYLRFKKSKRYSPQYILAYSFYM | 360 |
| ECO0411 | APMLNIYDGSFISSKLISEFGVIGALMCIFYFFYFSRFYLRFKKSKRYSPQYILAYSFYM | 360 |
| ECO0224 | APMLNIYDGSFISSKLISEFGVIGALMCIFYFFYFSRFYLRFKKSKRYSPQYILAYSFYM | 360 |
| ECO0433 | APMLNIYDGSFISSKLISEFGVIGALMCIFYFFYFSRFYLRFKKSKRYSPQYILAYSFYM | 360 |
| VREC0693 | APMLNIYDGSFISSKLISEFGVIGALMCIFYFFYFSRFYLRFKKSKRYSPQYILAYSFYM | 360 |
| VREC0829 | APMLNIYDGSFISSKLISEFGVIGALMCIFYFFYFSRFYLRFKKSKRYSPQYILAYSFYM | 360 |
| VREC0865 | APMLNIYDGSFISSKLISEFGVIGALMCIFYFFYFSRFYLRFKKSKRYSPQYILAYSFYM | 360 |
| VREC0926 | APMLNIYDGSFISSKLISEFGVIGALMCIFYFFYFSRFYLRFKKSKRYSPQYILAYSFYM | 360 |
| VREC1013 | APMLNIYDGSFISSKLISEFGVIGALMCIFYFFYFSRFYLRFKKSKRYSPQYILAYSFYM | 360 |
| VREC1073 | APMLNIYDGSFISSKLISEFGVIGALMCIFYFFYFSRFYLRFKKSKRYSPQYILAYSFYM | 360 |
| VREC1101 | APMLNIYDGSFISSKLISEFGVIGALMCIFYFFYFSRFYLRFKKSKRYSPQYILAYSFYM | 360 |
| VREC1106 | APMLNIYDGSFISSKLISEFGVIGALMCIFYFFYFSRFYLRFKKSKRYSPQYILAYSFYM | 360 |
| VREC1428 | APMLNIYDGSFISSKLISEFGVIGALMCIFYFFYFSRFYLRFKKSKRYSPQYILAYSFYM | 360 |

```
VREC1630        APMLNIYDGSFISSKLISEFGVIGALMCIFYFFYFSRFYLRFKKSKRYSPQYILAYSFYM        360
VRES0700        APMLNIYDGSFISSKLISEFGVIGALMCIFYFFYFSRFYLRFKKSKRYSPQYILAYSFYM        360
VRES1160        APMLNIYDGSFISSKLISEFGVIGALMCIFYFFYFSRFYLRFKKSKRYSPQYILAYSFYM        360
VREC0645        APMLNIYDGSFISSKLISEFGVIGALMCIFYFFYFSRFYLRFKKSKRYSPQYILAYSFYM        360
VRES0739        APMLNIYDGSFISSKLISEFGVIGALMCIFYFFYFSRFYLRFKKSKRYSPQYILAYSFYM        360
VRES1619        APMLNIYDGSFISSKLISEFGVIGALMCIFYFFYFSRFYLRFKKSKRYSPQYILAYSFYM        360
VRES1610        APMLNIYDGSFISSKLISEFGVIGALMCIFYFFYFSRFYLRFKKSKRYSPQYILAYSFYM        360
ECO0211         APMLNIYDGSFISSKLISEFGVIGALMCIFYFFYFSRFYLRFKKSKRYSPQYILAYSFYM        360
ECO0061         APMLNIYDGSFISSKLISEFGVIGALMCIFYFFYFSRFYLRFKKSKRYSPQYILAYSFYM        360
VREC1403        APMLNIYDGSFISSKLISEFGVIGALMCIFYFFYFSRFYLRFKKSKRYSPQYILAYSFYM        360
NCTC13441       APMLNIYDGSFISSKLISEFGVIGALMCIFYFFYFSRFYLRFKKSKRYSPQYILAYSFYM        360
ECO0218orf2     APMLNIYDGSFISSKLISEFGVIGALMCIFYFFYFSRFYLRFKKSKRYSPQYILAYSFYM        129
ECO0387orf2     APMLNIYDGSFISSKLISEFGVIGALMCIFYFFYFSRFYLRFKKSKRYSPQYILAYSFYM        160
ECO0218orf1     ------------------------------------------------------------        207
ECO0387orf1     ------------------------------------------------------------        193
```

```
ECO0353         CFFIPLFIRGAGYINPYVFMLFSSIFLCKYHAKNILMKSNVQIAI     405
ECO0431         CFFIPLFIRGAGYINPYVFMLFSSIFLCKYHAKNILMKSNVQIAI     405
ECO0411         CFFIPLFIRGAGYINPYVFMLFSSIFLCKYHAKNILMKSNVQIAI     405
ECO0224         CFFIPLFIRGAGYINPYVFMLFSSIFLCKYHAKNILMKSNVQIAI     405
ECO0433         CFFIPLFIRGAGYINPYVFMLFSSIFLCKYHAKNILMKSNVQIAI     405
VREC0693        CFFIPLFIRGAGYINPYVFMLFSSIFLCKYHAKNILMKSNVQIAI     405
VREC0829        CFFIPLFIRGAGYINPYVFMLFSSIFLCKYHAKNILMKSNVQIAI     405
VREC0865        CFFIPLFIRGAGYINPYVFMLFSSIFLCKYHAKNILMKSNVQIAI     405
VREC0926        CFFIPLFIRGAGYINPYVFMLFSSIFLCKYHAKNILMKSNVQIAI     405
VREC1013        CFFIPLFIRGAGYINPYVFMLFSSIFLCKYHAKNILMKSNVQIAI     405
VREC1073        CFFIPLFIRGAGYINPYVFMLFSSIFLCKYHAKNILMKSNVQIAI     405
VREC1101        CFFIPLFIRGAGYINPYVFMLFSSIFLCKYHAKNILMKSNVQIAI     405
VREC1106        CFFIPLFIRGAGYINPYVFMLFSSIFLCKYHAKNILMKSNVQIAI     405
VREC1428        CFFIPLFIRGAGYINPYVFMLFSSIFLCKYHAKNILMKSNVQIAI     405
VREC1630        CFFIPLFIRGAGYINPYVFMLFSSIFLCKYHAKNILMKSNVQIAI     405
VRES0700        CFFIPLFIRGAGYINPYVFMLFSSIFLCKYHAKNILMKSNVQIAI     405
VRES1160        CFFIPLFIRGAGYINPYVFMLFSSIFLCKYHAKNILMKSNVQIAI     405
VREC0645        CFFIPLFIRGAGYINPYVFMLFSSIFLCKYHAKNILMKSNVQIAI     405
VRES0739        CFFIPLFIRGAGYINPYVFMLFSSIFLCKYHAKNILMKSNVQIAI     405
VRES1619        CFFIPLFIRGAGYINPYVFMLFSSIFLCKYHAKNILMKSNVQIAI     405
VRES1610        CFFIPLFIRGAGYINPYVFMLFSSIFLCKYHAKNILMKSNVQIAI     405
ECO0211         CFFIPLFIRGAGYINPYVFMLFSSIFLCKYHAKNILMKSNVQIAI     405
ECO0061         CFFIPLFIRGAGYINPYVFMLFSSIFLCKYHAKNILMKSNVQIAI     405
VREC1403        CFFIPLFIRGAGYINPYVFMLFSSIFLCKYHAKNILMKSNVQIAI     405
NCTC13441       CFFIPLFIRGAGYINPYVFMLFSSIFLCKYHAKNILMKSNVQIAI     405
ECO0218orf2     CFFIPLFIRGAGYINPYVFMLFSSIFLCKYHAKNILMKSNVQIAI     174
ECO0387orf2     CFFIPLFIRGAGYINPYVFMLFSSIFLCKYHAKNILMKSNVQIAI     205
ECO0218orf1     ---------------------------------------------     207
ECO0387orf1     ---------------------------------------------     193
```

CLUSTAL O(1.2.4) multiple sequence alignment **RfaZ**

```
VREC0645_1      MKNIRYIDKKDVENLIENKTSDDVIIFLSGPTSQKTPLSVLRTKDIIAVNGSVQYLFKS- 59
VRES1160        MKNIRYIDKKDVENLIENKTSDDVIIFLSGPTSQKTPLSVLRTKDIIAVNGSVQYLLSHN 60
VREC0645_2      ------------------------------------------------MVLCNICLSHN 11
VRES0708        MKNIRYIDKKDVENLIENKTSDDVIIFLSGPTSQKTPLSVLRTKDIIAVNGSVQYLLSHN 60
ECO0216         MKNIRYIDKKDVENLIENKTSDDVIIFLSGPTSQKTPLSVLRTKDIIAVNGSVQYLLSHN 60
ECO0172         MKNIRYIDKKDVENLIENKTSDDVIIFLSGPTSQKTPLSVLRTKDIIAVNGSVQYLLSHN 60
ECO0056         MKNIRYIDKKDVENLIENKTSDDVIIFLSGPTSQKTPLSVLRTKDIIAVNGSVQYLLSHN 60
NCTC13441       MKNIRYIDKKDVENLIENKTSDDVIIFLSGPTSQKTPLSVLRTKDIIAVNGSVQYLLSHN 60
VRES1100        MKNIRYIDKKDVENLIENKTSDDVIIFLSGPTSQKTPLSVLRTKDIIAVNGSVQYLLSHN 60
VRES0739        MKNIRYIDKKDVENLIENKTSDDVIIFLSGPTSQKTPLSVLRTKDIIAVNGSVQYLLSHN 60
VRES0710        MKNIRYIDKKDVENLIENKTSDDVIIFLSGPTSQKTPLSVLRTKDIIAVNGSVQYLLSHN 60
ECO0257         MKNIRYIDKKDVENLIENKTSDDVIIFLSGPTSQKTPLSVLRTKDIIAVNGSVQYLLSHN 60
VRES1619        MKNIRYIDKKDVENLIENKTSDDVIIFLSGPTSQKTPLSVLRTKDIIAVNGSVQYLLSHN 60
VRES1610        MKNIRYIDKKDVENLIENKTSDDVIIFLSGPTSQKTPLSVLRTKDIIAVNGSVQYLLSHN 60
                                                                   :  :.
```

```
VREC0645_1      ------------------------------------------------------------ 59
VRES1160        IVPFIYVLTDVRFLHQRRDDFYKFSQRSRYTIVNVDVYEHASKEDKLYILQNCLVLRSFY 120
VREC0645_2      IVPFIYVLTDVRFLHQRRDDFYKFSQRSRYTIVNVDVYEHASKEDKLYILQNCLVLRSFY 71
VRES0708        IVPFIYVLTDVRFLHQRRDDFYKFSQRSRYTIVNVDVYEHASKEDKLYILQNCLVLRSFY 120
ECO0216         IVPFIYVLTDVRFLHQRRDDFYKFSQRSRYTIVNVDVYEHASKEDKLYILQNCLVLRSFY 120
ECO0172         IVPFIYVLTDVRFLHQRRDDFYKFSQRSRYTIVNVDVYEHASKEDKLYILQNCLVLRSFY 120
ECO0056         IVPFIYVLTDVRFLHQRRDDFYKFSQRSRYTIVNVDVYEHASKEDKLYILQNCLVLRSFY 120
```

```
NCTC13441       IVPFIYVLTDVRFLHQRRDDFYKFSQRSRYTIVNVDVYEHASKEDKLYILQNCLVLRSFY 120
VRES1100        IVPFIYVLTDVRFLHQRRDDFYKFSQRSRYTIVNVDVYEHASKEDKLYILQNCLVLRSFY 120
VRES0739        IVPFIYVLTDVRFLHQRRDDFYKFSQRSRYTIVNVDVYEHASKEDKLYILQNCLVLRSFY 120
VRES0710        IVPFIYVLTDVRFLHQRRDDFYKFSQRSRYTIVNVDVYEHASKEDKLYILQNCLVLRSFY 120
ECO0257         IVPFIYVLTDVRFLHQRRDDFYKFSQRSRYTIVNVDVYEHASKEDKLYILQNCLVLRSFY 120
VRES1619        IVPFIYVLTDVRFLHQRRDDFYKFSQRSRYTIVNVDVYEHASKEDKLYILQNCLVLRSFY 120
VRES1610        IVPFIYVLTDVRFLHQRRDDFYKFSQRSRYTIVNVDVYEHASKEDKLYILQNCLVLRSFY 120

VREC0645_1      ------------------------------------------------------------ 59
VRES1160        RREKGGFIKKIKFNILSQIHKELLISVPLSKKGRLVGFCKDISFGYCSCHTIAFAAIQIA 180
VREC0645_2      RREKGGFIKKIKFNILSQIHKELLISVPLSKKGRLVGFCKDISFGYCSCHTIAFAAIQIA 131
VRES0708        RREKGGFIKKIKFNILSQIHKELLISVPLSKKGRLVGFCKDISFGYCSCHTIAFAAIQIA 180
ECO0216         RREKGGFIKKIKFNILSQIHKELLISVPLSKKGRLVGFCKDISFGYCSCHTIAFAAIQIA 180
ECO0172         RREKGGFIKKIKFNILSQIHKELLISVPLSKKGRLVGFCKDISFGYCSCHTIAFAAIQIA 180
ECO0056         RREKGGFIKKIKFNILSQIHKELLISVPLSKKGRLVGFCKDISFGYCSCHTIAFAAIQIA 180
NCTC13441       RREKGGFIKKIKFNILSQIHKELLISVPLSKKGRLVGFCKDISFGYCSCHTIAFAAIQIA 180
VRES1100        RREKGGFIKKIKFNILSQIHKELLISVPLSKKGRLVGFCKDISFGYCSCHTIAFAAIQIA 180
VRES0739        RREKGGFIKKIKFNILSQIHKELLISVPLSKKGRLVGFCKDISFGYCSCHTIAFAAIQIA 180
VRES0710        RREKGGFIKKIKFNILSQIHKELLISVPLSKKGRLVGFCKDISFGYCSCHTIAFAAIQIA 180
ECO0257         RREKGGFIKKIKFNILSQIHKELLISVPLSKKGRLVGFCKDISFGYCSCHTIAFAAIQIA 180
VRES1619        RREKGGFIKKIKFNILSQIHKELLISVPLSKKGRLVGFCKDISFGYCSCHTIAFAAIQIA 180
VRES1610        RREKGGFIKKIKFNILSQIHKELLISVPLSKKGRLVGFCKDISFGYCSCHTIAFAAIQIA 180

VREC0645_1      ------------------------------------------------------------ 59
VRES1160        YSLKYARIICSGLDLTGNCSRFYDENNNPMPSELSKDLFKILPFFRFMRDNVEDINIYNL 240
VREC0645_2      YSLKYARIICSGLDLTGNCSRFYDENNNPMPSELSKDLFKILPFFRFMRDNVEDINIYNL 191
VRES0708        YSLKYARIICSGLDLTGNCSRFYDENNNPMPSELSKDLFKILSFFRFMRDNVEDINIYNL 240
ECO0216         YSLKYARIICSGLDLTGNCSRFYDENNNPMPSELSKDLFKILPFFRFMRDNVEDINIYNL 240
ECO0172         YSLKYARIICSGLDLTGNCSRFYDENNNPMPSELSKDLFKILPFFRFMRDNVEDINIYNL 240
ECO0056         YSLKYARIICSGLDLTGNCSRFYDENNNPMPSELSKDLFKILPFFRFMRDNVEDINIYNL 240
NCTC13441       YSLKYARIICSGLDLTGNCSRFYDENNNPMPSELSKDLFKILPFFRFMRDNVEDINIYNL 240
VRES1100        YSLKYARIICSGLDLTGNCSRFYDENNNPMPSELSKDLFKILPFFRFMRDNVEDINIYNL 240
VRES0739        YSLKYARIICSGLDLTGNCSRFYDENNNPMPSELSKDLFKILPFFRFMRDNVEDINIYNL 240
VRES0710        YSLKYARIICSGLDLTGNCSRFYDENNNPMPSELSKDLFKILPFFRFMRDNVEDINIYNL 240
ECO0257         YSLKYARIICSGLDLTGNCSRFYDENNNPMPSELSKDLFKILPFFRFMRDNVEDINIYNL 240
VRES1619        YSLKYARIICSGLDLTGNCSRFYDENNNPMPSKLSKDLFKILPFFRFMRDNVEDINIYNL 240
VRES1610        YSLKYARIICSGLDLTGNCSRFYDENNNPMPSKLSKDLFKILPFFRFMRDNVEDINIYNL 240

VREC0645_1      ------------------------------------------------------------ 59
VRES1160        SDDTAISYDVIPFIKFQDISREESKDKTRKKCNIELQLILMLINHPETKVIWYKNAFRNF 300
VREC0645_2      SDDTAISYDVIPFIKFQDISREESKDKTRKKMQYRTSTDSYA------------N----- 234
VRES0708        SDDTAISYDVIPFIKFQDISREESKDKTRKKMQYRTSTDSYA------------N----- 283
ECO0216         SDDTAISYDVIPFIKFQDISREESKDKTRKKMQYRTSTDSYA------------N----- 283
ECO0172         SDDTAISYDVIPFIKFQDISREESKDKTRKKMQYRTSTDSYA------------N----- 283
ECO0056         SDDTAISYDVIPFIKFQDISREESKDKTRKKMQYRTSTDSYA------------N----- 283
NCTC13441       SDDTAISYDVIPFIKFQDISREESKDKTRKKMQYRTSTDSYA------------N----- 283
VRES1100        SDDTAISYDVIPFIKFQDISREESKDKTRKKMQYRTSTDSYA------------N----- 283
VRES0739        SDDTAISYDVIPFIKFQDISREESKDKTRKKMQYRTSTDSYA------------N----- 283
VRES0710        SDDTAISYDVIPFIKFQDISREESKDKTRKKMQYRTSTDSYA------------N----- 283
ECO0257         SDDTAISYDVIPFIKFQDISREESKDKTRKKMQYRTSTDSYA------------N----- 283
VRES1619        SDDTAISYDVIPFIKFQDISREESKDKTRKKMQYRTSTDSYA------------N----- 283
VRES1610        SDDTAISYDVIPFIKFQDISREESKDKTRKKMQYRTSTDSYA------------N----- 283

VREC0645_1      -------------        59
VRES1160        SQKEKILYKQNKN        313
VREC0645_2      -------------        234
VRES0708        -------------        283
ECO0216         -------------        283
ECO0172         -------------        283
ECO0056         -------------        283
NCTC13441       -------------        283
VRES1100        -------------        283
VRES0739        -------------        283
VRES0710        -------------        283
ECO0257         -------------        283
VRES1619        -------------        283
VRES1610        -------------        283
```

CLUSTAL O(1.2.4) multiple sequence alignment **RfaJ**

```
ECO0216         MDSFPAIEIDKVKAWDFRLANINTSECLNVAYGVDANYLDGVGVSITSIVLNNRHINLDF 60
ECO0172         MDSFPAIEIDKVKAWDFRLANINTSECLNVAYGVDANYLDGVGVSITSIVLNNRHINLDF 60
ECO0056         MDSFPAIEIDKVKAWDFRLANINTSECLNVAYGVDANYLDGVGVSITSIVLNNRHINLDF 60
NCTC13441       MDSFPAIEIDKVKAWDFRLANINTSECLNVAYGVDANYLDGVGVSITSIVLNNRHINLDF 60
VRES1619        MDSFPAIEIDKVKAWDFRLANINTSECLNVAYGVDANYLDGVGVSITSIVLNNRHINLDF 60
VRES1610        MDSFPAIEIDKVKAWDFRLANINTSECLNVAYGVDANYLDGVGVSITSIVLNNRHINLDF 60
VRES1160        MDSFPAIEIDKVKAWDFRLANINTSECLNVAYGVDANYLDGVGVSITSIVLNNRHINLDF 60
VRES1100        MDSFPAIEIDKVKAWDFRLANINTSECLNVAYGVDANYLDGVGVSITSIVLNNRHINLDF 60
VRES0739        MDSFPAIEIDKVKAWDFRLANINTSECLNVAYGVDANYLDGVGVSITSIVLNNRHINLDF 60
VRES0710        MDSFPAIEIDKVKAWDFRLANINTSECLNVAYGVDANYLDGVGVSITSIVLNNRHINLDF 60
VRES0708        MDSFPAIEIDKVKAWDFRLANINTSECLNVAYGVDANYLDGVGVSITSIVLNNRHINLDF 60
VREC0645        MDSFPAIEIDKVKAWDFRLANINTSECLNVAYGVDANYLDGVGVSITSIVLNNRHINLDF 60
ECO0257         MDSFPAIEIDKVKAWDFRLANINTSECLNVAYGVDANYLDGVGVSITSIVLNNRHINLDF 60
                ************************************************************

ECO0216         YIIADVYNDAFFQKVAKLAEQYQLRITLYRINTDKLQCLPCTQVWSRAMYFRLFAFQLLG 120
ECO0172         YIIADVYNDAFFQKVAKLAEQYQLRITLYRINTDKLQCLPCTQVWSRAMYFRLFAFQLLG 120
ECO0056         YIIADVYNDAFFQKVAKLAEQYQLRITLYRINTDKLQCLPCTQVWSRAMYFRLFAFQLLG 120
NCTC13441       YIIADVYNDAFFQKVAKLAEQYQLRITLYRINTDKLQCLPCTQVWSRAMYFRLFAFQLLG 120
VRES1619        YIIADVYNDAFFQKVAKLAEQYQLRITLYRINTDKLQCLPCTQVWSRAMYFRLFAFQLLG 120
VRES1610        YIIADVYNDAFFQKVAKLAEQYQLRITLYRINTDKLQCLPCTQVWSRAMYFRLFAFQLLG 120
VRES1160        YIIADVYNDAFFQKVAKLAEQYQLRITLYRINTDKLQCLPCTQVWSRAMYFRLFAFQLLG 120
VRES1100        YIIADVYNDAFFQKVAKLAEQYQLRITLYRINTDKLQCLPCTQVWSRAMYFRLFAFQLLG 120
VRES0739        YIIADVYNDAFFQKVAKLAEQYQLRITLYRINTDKLQCLPCTQVWSRAMYFRLFAFQLLG 120
VRES0710        YIIADVYNDAFFQKVAKLAEQYQLRITLYRINTDKLQCLPCTQVWSRAMYFRLFAFQLLG 120
VRES0708        YIIADVYNDAFFQKVAKLAEQYQLRITLYRINTDKLQCLPCTQVWSRAMYFRLFAFQLLG 120
VREC0645        YIIADVYNDAFFQKVAKLAEQYQLRITLYRINTDKLQCLPCTQVWSRAMYFRLFAFQLLG 120
ECO0257         YIIADVYNDAFFQKVAKLAEQYQLRITLYRINTDKLQCLPCTQVWSRAMYFRLFAFQLLG 120
                ************************************************************

ECO0216         LTLDRLLYLDADVVCKGDISQLLHLDLNGAVAAVVKDVDPMQEKAASRLSDPELLGQYFN 180
ECO0172         LTLDRLLYLDADVVCKGDISQLLHLDLNGAVAAVVKDVDPMQEKAASRLSDPELLGQYFN 180
ECO0056         LTLDRLLYLDADVVCKGDISQLLHLDLNGAVAAVVKDVDPMQEKAASRLSDPELLGQYFN 180
NCTC13441       LTLDRLLYLDADVVCKGDISQLLHLDLNGAVAAVVKDVDPMQEKAASRLSDPELLGQYFN 180
VRES1619        LTLDRLLYLDADVVCKGDISQLLHLDLNGAVAAVVKDVDPMQEKAASRLSDPELLGQYFN 180
VRES1610        LTLDRLLYLDADVVCKGDISQLLHLDLNGAVAAVVKDVDPMQEKAASRLSDPELLGQYFN 180
VRES1160        LTLDRLLYLDADVVCKGDISQLLHLDLNGAVAAVVKDVDPMQEKAASRLSDPELLGQYFN 180
VRES1100        LTLDRLLYLDADVVCKGDISQLLHLDLNGAVAAVVKDVDPMQEKAASRLSDPELLGQYFN 180
VRES0739        LTLDRLLYLDADVVCKGDISQLLHLDLNGAVAAVVKDVDPMQEKAASRLSDPELLGQYFN 180
VRES0710        LTLDRLLYLDADVVCKGDISQLLHLDLNGAVAAVVKDVDPMQEKAASRLSDPELLGQYFN 180
VRES0708        LTLDRLLYLDADVVCKGDISQLLHLDLNGAVAAVVKDVDPMQEKAASRLSDPELLGQYFN 180
VREC0645        LTLDRLLYLDADVVCKGDISQLLHLDLNGAVAAVVKDVDPMQEKAASRLSDPELLGQYFN 180
ECO0257         LTLDRLLYLDADVVCKGDISQLLHLDLNGAVAAVVKDVDPMQEKAASRLSDPELLGQYFN 180
                ************************************************************

ECO0216         SCVVYLDLKKWANAKLTEKALSILMSKDNVYKYPDQDVMNVLLKGMTIFLPRGYNTIYTI 240
ECO0172         SGVVYLDLKKWANAKLTEKALSILMSKDNVYKYPDQDVMNVLLKGMTIFLPRGYNTIYTI 240
ECO0056         SGVVYLDLKKWANAKLTEKALSILMSKDNVYKYPDQDVMNVLLKGMTIFLPRGYNTIYTI 240
NCTC13441       SGVVYLDLKKWANAKLTEKALSILMSKDNVYKYPDQDVMNVLLKGMTIFLPRGYNTIYTI 240
VRES1619        SGVVYLDLKKWANAKLTEKALSILMSKDNVYKYPDQDVMNVLLKGMTIFLPRGYNTIYTI 240
VRES1610        SGVVYLDLKKWANAKLTEKALSILMSKDNVYKYPDQDVMNVLLKGMTIFLPRGYNTIYTI 240
VRES1160        SGVVYLDLKKWANAKLTEKALSILMSKDNVYKYPDQDVMNVLLKGMTIFLPRGYNTIYTI 240
VRES1100        SGVVYLDLKKWANAKLTEKALSILMSKDNVYKYPDQDVMNVLLKGMTIFLPRGYNTIYTI 240
VRES0739        SGVVYLDLKKWANAKLTEKALSILMSKDNVYKYPDQDVMNVLLKGMTIFLPRGYNTIYTI 240
VRES0710        SGVVYLDLKKWANAKLTEKALSILMSKDNVYKYPDQDVMNVLLKGMTIFLPRGYNTIYTI 240
VRES0708        SGVVYLDLKKWANAKLTEKALSILMSKDNVYKYPDQDVMNVLLKGMTIFLPRGYNTIYTI 240
VREC0645        SGVVYLDLKKWANAKLTEKALSILMSKDNVYKYPDQDVMNVLLKGMTIFLPRGYNTIYTI 240
ECO0257         SGVVYLDLKKWANAKLTEKALSILMSKDNVYKYPDQDVMNVLLKGMTIFLPRGYNTIYTI 240
                * **********************************************************

ECO0216         KSELKDKTHQNYKKLIIENTLLIHYTGATKPWHKWAIYPSTKYYKIALDNSPWKDDSPRD 300
ECO0172         KSELKDKTHQNYKKLIIENTLLIHYTGATKPWHKWAIYPSTKYYKIALDNSPWKDDSPRD 300
ECO0056         KSELKDKTHQNYKKLIIENTLLIHYTGATKPWHKWAIYPSTKYYKIALDNSPWKDDSPRD 300
NCTC13441       KSELKDKTHQNYKKLIIENTLLIHYTGATKPWHKWAIYPSTKYYKIALDNSPWKDDSPRD 300
VRES1619        KSELKDKTHQNYKKLIIENTLLIHYTGATKPWHKWAIYPSTKYYKIALDNSPWKDDSPRD 300
VRES1610        KSELKDKTHQNYKKLIIENTLLIHYTGATKPWHKWAIYPSTKYYKIALDNSPWKDDSPRD 300
VRES1160        KSELKDKTHQNYKKLIIENTLLIHYTGATKPWHKWAIYPSTKYYKIALDNSPWKDDSPRD 300
VRES1100        KSELKDKTHQNYKKLIIENTLLIHYTGATKPWHKWAIYPSTKYYKIALDNSPWKDDSPRD 300
VRES0739        KSELKDKTHQNYKKLIIENTLLIHYTGATKPWHKWAIYPSTKYYKIALDNSPWKDDSPRD 300
VRES0710        KSELKDKTHQNYKKLIIENTLLIHYTGATKPWHKWAIYPSTKYYKIALDNSPWKDDSPRD 300
VRES0708        KSELKDKTHQNYKKLIIENTLLIHYTGATKPWHKWAIYPSTKYYKIALDNSPWKDDSPRD 300
VREC0645        KSELKDKTHQNYKKLIIENTLLIHYTGATKPWHKWAIYPSTKYYKIALDNSPWKDDSPRD 300
ECO0257         KSELKDKTHQNYKKLIIENTLLIHYTGATKPWHKWAIYPSTKYYKIALDNSPWKDDSPRD 300
                ************************************************************
```

```
ECO0216        AKSIIEFKKRYKHLLVQHHYISGIIAGVCYLCRKYYPK    338
ECO0172        AKSIIEFKKRYKHLLVQHHYISGIIAGVCYLCRKYYPK    338
ECO0056        AKSIIEFKKRYKHLLVQHHYISGIIAGVCYLCRKYYPK    338
NCTC13441      AKSIIEFKKRYKHLLVQHHYISGIIAGVCYLCRKYYPK    338
VRES1619       AKSIIEFKKRYKHLLVQHHYISGIIAGVCYLCRKYYPK    338
VRES1610       AKSIIEFKKRYKHLLVQHHYISGIIAGVCYLCRKYYPK    338
VRES1160       AKSIIEFKKRYKHLLVQHHYISGIIAGVCYLCRKYYPK    338
VRES1100       AKSIIEFKKRYKHLLVQHHYISGIIAGVCYLCRKYYPK    338
VRES0739       AKSIIEFKKRYKHLLVQHHYISGIIAGVCYLCRKYYPK    338
VRES0710       AKSIIEFKKRYKHLLVQHHYISGIIAGVCYLCRKYYPK    338
VRES0708       AKSIIEFKKRYKHLLVQHHYISGIIAGVCYLCRKYYPK    338
VREC0645       AKSIIEFKKRYKHLLVQHHYISGIIAGVCYLCRKYYPK    338
ECO0257        AKSIIEFKKRYKHLLVQHHYISGIIAGVCYLCRKYYPK    338
               **************************************
```

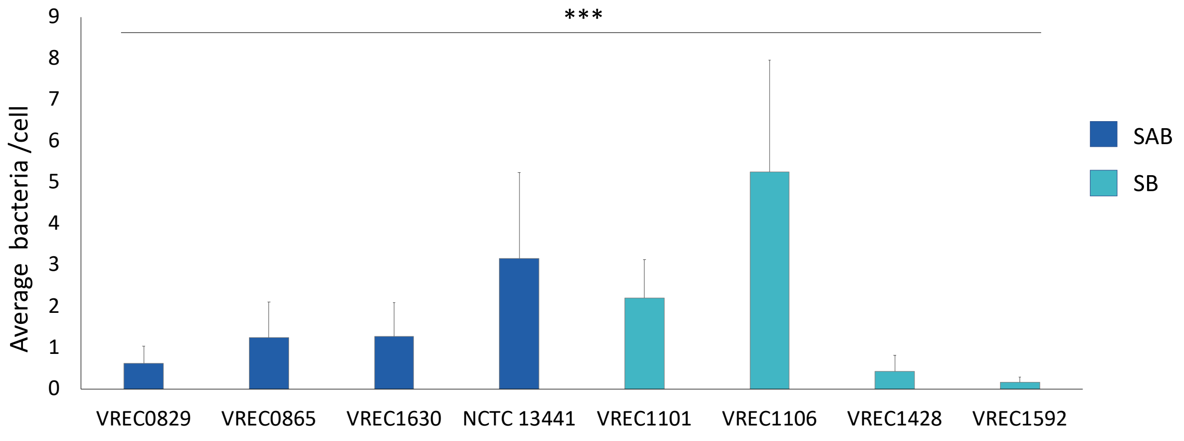


**Supplementary Figure S6: Background differences in phagocytosis for isolates without antibody present.** Macrophages were stained with CellMasks Orange (cytoplasm) and DAPI (nucleic acid) and bacteria were visualised using KM467 labelled with Alexa Fluor 647. Images were obtained using a 40x air objective on the Opera Phenix. Images were analysed in Harmony using predefined building blocks to segment nuclei and cytoplasm to define the number of cells, and to count individual bacteria (using Find Spots) within the cells. Error bars represent standard deviation of 3 replicates. Significance was determined by ANOVA test (***<0.001).

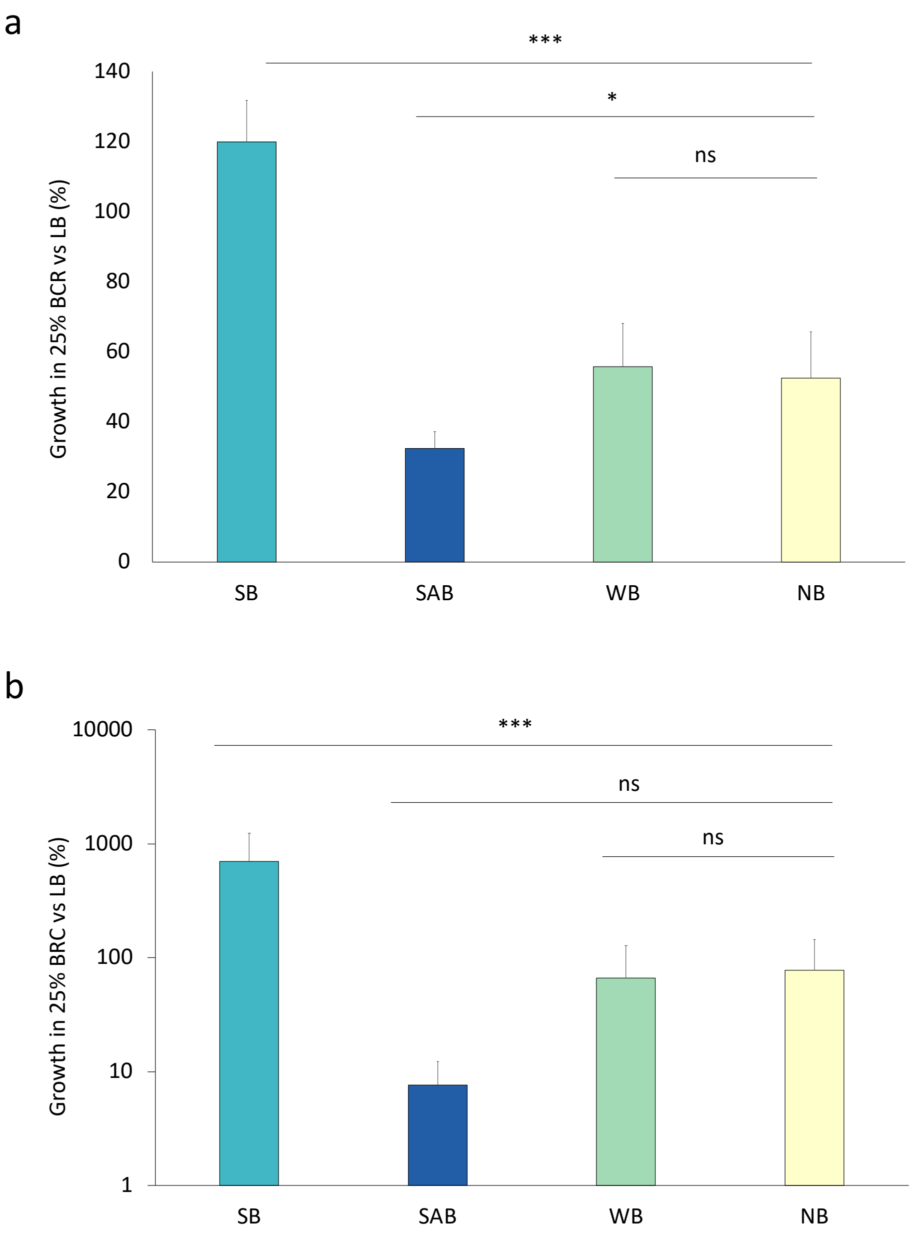


**Supplementary Figure S7: Serum Resistance Reproducibility.** Growth in LB broth in the presence or absence of 25% BRC was measured by OD600 (**a**) or by cfu counting (**b**) after 4 hours and plotted as percentage growth in the presence of serum compared to LB alone. The average of representative isolates per phenotype (SB=4, SAB=4, WB=3, NB =3) of 3 replicates is shown, and error bars represent standard deviation. Significance was determined by ANOVA, Tukey's post-hoc (*<0.05, ** <0.01, *** <0.001).

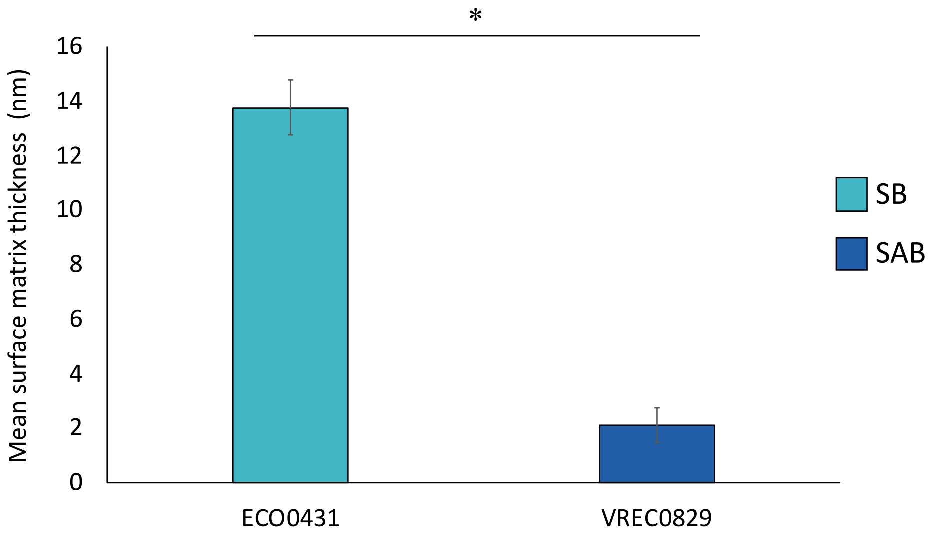


**Supplementary Figure S8: Differences in surface matrix thickness.** The mean surface matrix thickness of ECO0431 (SB, serum resistant) and VREC0829 (SAB, serum sensitive) from 20 measurements across 10 individual bacteria. Error bars represent standard deviation and significance was determined by t-test (*<0.05, ** <0.01, *** <0.001).

## Supplementary Table 2: Antibody binding analysis pipeline (Harmony v4.8)

| | | | |
|---|---|---|---|
| **Input Image** | Input | | |
| | **Flatfield Correction**: Basic Brightfield Correction<br>**Stack Processing**: Individual Planes<br>Create Global Image<br>**Min. Global Binning**: Dynamic | | |
| **Find Spots** | Input | Method | Output |
| | **Channel**: DAPI<br>**ROI**: None | **Method**: D<br>Detection Sensitivity: 0.5<br>Splitting Sensitivity: 0.5<br>Background correction: 0.5<br>Calculate Spot Properties | Output Population: Spots |
| **Calculate Morphology Properties** | Input | Method | Output |
| | **Population**: Spots<br>**Region**: Spot | **Method**: Standard<br>Area<br>Length | Property Prefix: Spot morphology |
| **Select Population** | Input | Method | Output |
| | **Population**: Spots | **Method**: Filter by Property<br>Spot morphology Length[µm]: <5<br>Spot morphology area [µm$^2$]: >0.7<br>Boolean Operations: F1 and F2 | Output Population: Spots Selected |
| **Select Population (2)** | Input | Method | Output |
| | **Population**: Spots Selected | Method: Common Filters<br>Remove Border Objects<br>Region: Spot | Output Population: Spots Selected no border |
| **Select Region** | Input | Method | Output |
| | **Population**: Spots Selected no border<br>**Region**: Spot | **Method**: Resize Region [µm/px]<br>Outer Border: -0.5µm<br>Inner Border: INF µm | Output Population: final bacteria |
| **Calculate Intensity Properties** | Input | Method | Output |
| | **Channel**: Alexa 647<br>**Population**: Spots Selected no border<br>**Region**: final bacteria | **Method**: Standard<br>Mean<br>Standard Deviation<br>Coefficient of Variance<br>Median<br>Maximum<br>Minimum<br>Contrast | Property Prefix: Intensity final bacteria Alexa 647 |
| **Select Population (3)** | Input | Method | Output |
| | **Population**: Spots Selected no border | **Method**: Filter by Property<br>Intensity final bacteria Alexa 647 Mean: > 600 | Output Population: Strong ALEXA |
| **Select Population (4)** | Input | Method | Output |

| | | | |
|---|---|---|---|
| | **Population**: Spots Selected no border | **Method**: Filter by Property<br>Intensity final bacteria Alexa 647 Mean: < 599<br>Intensity final bacteria Alexa 647 Mean: > 180 | Output Population: Weak ALEXA |
| **Select Population (5)** | Input | Method | Output |
| | **Population**: Spots Selected no border | **Method**: Filter by Property<br>Intensity final bacteria Alexa 647 Mean: > 180 | Output Population: Total ALEXA |
| **Calculate Position Properties** | Input | Method | Output |
| | **Population**: Spots Selected no border<br>**Region**: final bacteria | **Method**: Standard<br>Nearest Neighbour Distance | Property Prefix: final bacteria nearest neighbour distance |
| **Select Population (6)** | Input | Method | Output |
| | **Population**: Spots Selected no border | **Method**: Filter by Property<br>: Filter by Property<br>Final bacteria nearest neighbour distance Nearest Neighbour Distance [µm] < 1 | Output Population: Neighbouring bacteria |
| **Modify Population** | Input | Method | Output |
| | **Population**: Neighbouring bacteria<br>**Region**: final bacteria | **Method**: Cluster by Distance<br>Distance: 0.2 µm<br>Area: > 2 $\mu m^2$ | Output Population: Clustered bacteria by distance<br>Output Region: Cluster of bacteria by distance |
| **Calculate Properties** | Input | Method | Output |
| | **Population**: Clustered bacteria by distance | **Method**: By Related Population<br>Related Population: Neighbouring bacteria<br>Number of Neighbouring bacteria | Property Suffix: bacteria in clusters |
| **Select Population (7)** | Input | Method | Output |
| | **Population**: Clustered bacteria by distance | **Method**: Filter by Property<br>Number of Neighbouring bacteria – bacteria in clusters: > 4 | Output Population: Cluster >4 bacteria |
| **Calculate Properties (2)** | Input | Method | Output |
| | **Population**: Cluster >4 bacteria | **Method**: By Related Population<br>Related Population: Neighbouring bacteria<br>Number of Neighbouring bacteria | Property Suffix: bacteria per real cluster |
| **Calculate Morphology Properties (2)** | Input | Method | Output |
| | **Population**: Clustered bacteria by distance<br>**Region**: Cluster of bacteria by distance | **Method**: Standard<br>Area | Property Prefix: area cluster of bacteria |
| **Select Population (8)** | Input | Method | Output |
| | **Population**: Clustered bacteria by distance | Method: Filter by Property<br>area cluster of bacteria Area [µm2]: >13 | Output Population: Agglutinated bacteria |
| **Calculate Properties (3)** | Input | Method | Output |
| | **Population**: Agglutinated by area | **Method**: By Related Population<br>Related Population: Neighbouring bacteria<br>Number of Neighbouring bacteria | Property Suffix: number of agglutinated bacteria |

## Well Results: list of outputs

**Population: Spots Selected no border**
Number of Objects
Relative Spot Intensity: Mean per Well
Corrected Spot Intensity: Mean per Well
Uncorrected Spot Intensity: Mean per Well
Spot Contrast: Mean per Well
Spot Background Intensity: Mean per Well
Spot Area [$px^2$]: Mean per Well
Region Intensity: Mean per Well
Spot to Region Intensity: Mean per Well
Spot morphology Area [μm2]: Mean per Well
Spot morphology Length [μm]: Mean per Well
Intensity final bacteria Alexa 647 Mean: Mean per Well
Intensity final bacteria Alexa 647 StdDev: Mean per Well
Intensity final bacteria Alexa 647 Median: Mean per Well
Intensity final bacteria Alexa 647 Maximum: Mean per Well
Intensity final bacteria Alexa 647 Minimum: Mean per Well
Intensity final bacteria Alexa 647 Su: Mean per Well
Intensity final bacteria Alexa 647 CV [%]: Mean per Well
Intensity final bacteria Alexa 647 Contrast: Mean per Well
Strong ALEXA: Mean per Well
Weak ALEXA: Mean per Well
Total ALEXA: Mean per Well
final bacteria nearest neighbour distance Nearest Neighbour Distance [μm]: Mean per Well
Neighbouring bacteria: Mean per Well

**Population: Strong ALEXA**
Number of Objects

**Population: Weak ALEXA**
Number of Objects

**Population: Total ALEXA**
Number of Objects

**Population: Clustered bacteria by distance**
Number of Neighbouring bacteria – bacteria in clusters: Mean per Well
Cluster >4 bacteria: Mean per Well
area cluster of bacteria Area [μm2]: Mean per Well
Agglutinated by area: mean and standard deviation

**Population: Cluster >4 bacteria**
Number of Neighbouring bacteria – bacteria in clusters: Mean per Well
Number of Neighbouring bacteria – bacteria per real cluster: Mean per Well

**Population: Agglutinated by area**
Number of Neighbouring bacteria – bacteria in clusters: Mean per Well
Cluster >4 bacteria: Mean per Well
area cluster of bacteria Area [μm2]: Mean per Well
Number of Neighbouring bacteria – number of agglutinating bacteria: Mean per Well

### Formula Outputs:

Formula: 100*(a/b)
Population a: Cluster >4 bacteria – Number of Neighbouring bacteria – bacteria per real cluster Sum
Population b: Spots Selected no border – Number of Objects
**% clustering**

Formula: 100*(a/b)
Population a: Agglutinated by area – Number of Neighbouring bacteria – number of agglutinated bacteria Sum
Population b: Spots Selected no border – Number of Objects
**% agglutination**

Formula: 100*(a/b)
Population a: Total ALEXA – Number of Objects
Population b: Spots Selected no border – Number of Objects
**% total binding**

Formula: 100*(a/b)
Population a: Strong ALEXA – Number of Objects
Population b: Spots Selected no border – Number of Objects
**% strong binding**

Formula: 100*(a/b)
Population a: Weak ALEXA – Number of Objects
Population b: Spots Selected no border – Number of Objects
**% weak binding**

## Supplementary Table 5: Macrophage Phagocytosis Pipeline (Harmony v4.8)

| | | | |
|---|---|---|---|
| **Input Image** | **Input** | | |
| | **Flatfield Correction: None, Brightfield Correction**<br>**Stack Processing: Maximum Projection**<br>**Min. Global Binning: Dynamic** | | |
| **Find Nuclei** | **Input** | **Method** | **Output** |
| | **Channel: DAPI**<br>**ROI: None** | **Method: B**<br>**Common Threshold: 0.4**<br>**Area: > 30um²**<br>**Splitting Coefficient: 7**<br>**Individual Threshold: 0.4**<br>**Contrast: > 0.1** | **Output Population: CELL** |
| **Find Cytoplasm** | **Input** | **Method** | **Output** |
| | **Channel: MitoTracker Orange**<br>**Nuclei: CELL** | **Method: D**<br>**Individual Threshold: 0.15** | |
| **Select Cell Region** | **Input** | **Method** | **Output** |
| | **Population: CELL** | **Method: Resize Region (%)**<br>**Region Type: Cell Region**<br>**Outer Border: 1[illegible]**<br>**Inner Border: 100%** | **Output Region: Cell Region** |
| **Find Spots** | **Input** | **Method** | **Output** |
| | **Channel: Alexa 647**<br>**ROI: CELL**<br>**ROI Region: Cell** | **Method: B**<br>**Detection Sensitivity: 0.5**<br>**Splitting Sensitivity: 0.5**<br>**Calculate Spot Properties** | **Output Population: intracellular bacteria** |

**Results:**

**Method: List of outputs**
**Population: CELL**
**Number of objects**

**Method: Standard Output**
**CELL – Number of Objects: Object Count**
**Output Name: CELL – Number of Objects**

**Method: Standard Output**
**Intracellular bacteria – Number of Objects: Object Count**
**Output Name: intracellular bacteria – Number of Objects**

**Method: Formula Output**
**Formula: a/b**
**Population Type: Objects**
**Variable a: intracellular bacteria – Number of Objects**
**Variable b: CELL – Number of Objects**
**Output: Number of intracellular bacteria per cell**
